# Pathology of Coronavirus Infections: A Review of Lesions in Animals in the One-Health Perspective

**DOI:** 10.3390/ani10122377

**Published:** 2020-12-11

**Authors:** Valentina Zappulli, Silvia Ferro, Federico Bonsembiante, Ginevra Brocca, Alessandro Calore, Laura Cavicchioli, Cinzia Centelleghe, Giorgia Corazzola, Steffen De Vreese, Maria Elena Gelain, Sandro Mazzariol, Valentina Moccia, Nicolò Rensi, Alessandro Sammarco, Filippo Torrigiani, Ranieri Verin, Massimo Castagnaro

**Affiliations:** 1 Department of Comparative Biomedicine and Food Science, University of Padua, Legnaro, 35020 Padua, Italy; valentina.zappulli@unipd.it (V.Z.); federico.bonsembiante@unipd.it (F.B.); ginevra.brocca@phd.unipd.it (G.B.); alessandro.calore.1@studenti.unipd.it (A.C.); laura.cavicchioli@unipd.it (L.C.); cinzia.centelleghe@unipd.it (C.C.); giorgia.corazzola@gmail.com (G.C.); steffen.devreese@studenti.unipd.it (S.D.V.); mariaelena.gelain@unipd.it (M.E.G.); sandro.mazzariol@unipd.it (S.M.); valentina.moccia@phd.unipd.it (V.M.); nicolo.rensi@phd.unipd.it (N.R.); alessandro.sammarco@unipd.it (A.S.); filippo.torrigiani@unipd.it (F.T.); ranieri.verin@unipd.it (R.V.); massimo.castagnaro@unipd.it (M.C.); 2 Department of Animal Medicine, Productions and Health, University of Padua, Legnaro, 35020 Padua, Italy; 3 Laboratory of Applied Bioacoustics, Technical University of Catalunya, BarcelonaTech, Vilanova i la Geltrù, 08800 Barcelona, Spain; 4 Department of Neurology and Radiology, Massachusetts General Hospital, Harvard Medical School, Boston, MA 02129, USA

**Keywords:** coronavirus, pathology, veterinary medicine, One Health

## Abstract

**Simple Summary:**

Coronaviruses are worldwide distributed RNA-viruses affecting several species, causing a broad spectrum of diseases with a zoonotic potential and the ability to jump from one host species to a different one, including humans. In the perspective of ‘One Health’ and the well-known recent Coronavirus-associated epidemics and pandemic, the aim of this review is to list all the animal species affected by Coronaviruses and to describe the lesions and the target organs. Information is given on the pathogenesis and the gross and histological lesions of pets, ferrets, bovines, sheep, goats, equine, swine, wild animals, non-human primates, marine mammals, laboratory animals, fish, reptiles, amphibian, and, briefly, humans.

**Abstract:**

Coronaviruses (CoVs) are worldwide distributed RNA-viruses affecting several species, including humans, and causing a broad spectrum of diseases. Historically, they have not been considered a severe threat to public health until two outbreaks of COVs-related atypical human pneumonia derived from animal hosts appeared in 2002 and in 2012. The concern related to CoVs infection dramatically rose after the COVID-19 global outbreak, for which a spill-over from wild animals is also most likely. In light of this CoV zoonotic risk, and their ability to adapt to new species and dramatically spread, it appears pivotal to understand the pathophysiology and mechanisms of tissue injury of known CoVs within the “One-Health” concept. This review specifically describes all CoVs diseases in animals, schematically representing the tissue damage and summarizing the major lesions in an attempt to compare and put them in relation, also with human infections. Some information on pathogenesis and genetic diversity is also included. Investigating the lesions and distribution of CoVs can be crucial to understand and monitor the evolution of these viruses as well as of other pathogens and to further deepen the pathogenesis and transmission of this disease to help public health preventive measures and therapies.

## 1. Introduction

In December 2019, numerous cases of viral interstitial pneumonia started to be diagnosed in people in China in Wuhan, Hubei province [1], after which the infection spread in many countries. On 30 January 2020, the World Health Organization (WHO) classified the global outbreak as a “public health emergency of international concern” [2], forcing all affected countries to take preventive measures in order to limit the spread of the infection. In the perspective of the One-Health concept, human lives are in a constant relationship with animals including pets, production animals, and wildlife. The interface humans–animals and the different environments shared are indeed a source of diseases that could impact strongly on public health as well as on social and economic levels, as we are currently experiencing during the recent pandemic event, even if not only restricted to the latter. Historically, the use of antibiotics and the introduction of vaccine campaigns seemed to control recurrent infectious disease outbreaks. Nevertheless, as a concomitant effect, not only has antibiotic resistance increased but there has been a substantial emergence of diseases, mainly of viral origin, from wildlife to humans, occasionally causing fatal outbreaks and pandemics [3,4,5]. For this reason, much effort has been put in, since then, by the scientific community in order to better understand, during the current pandemic, the specific etiological agent involved, the pathophysiology of the infection, the therapeutic responses and the best measures to confine the outbreaks. The agent isolated from pneumonia cases was classified as belonging to the *Coronaviridae* family, initially classified as 2019 novel coronavirus (nCoV) and subsequently renamed as Severe Acute Respiratory Syndrome (SARS)-CoV-2. As of 8 October 2020, the WHO reported that 235 countries were affected by SARC-CoV-2-associated disease (COVID-19), with 35,897,739 confirmed cases and a total of 1,048,781 confirmed deaths [6].

Historically, CoVs were not considered a severe threat to public health until two outbreaks of atypical pneumonia appeared in the recent past. The first, in 2002—later renamed SARS and caused by SARS-CoV—associated with high rates of fatalities, reaching up to 10% [2]. The second, ten years later, named Middle East Respiratory Syndrome (MERS), from the geographical area of the first isolation and caused by another pathogenic CoV (MERS-CoV), had a fatality rate up to 37% [2].

CoVs are well-known by the scientific community and particularly by veterinarians, as they can cause a wide range of diseases, mainly affecting the respiratory, gastro-intestinal, and central nervous systems [7], in a large number of host species, from birds to mammals, including humans [8].

CoVs are positive single-strand enveloped RNA viruses (+ssRNA) [9] with the largest genome (27–32 Kb) among all RNA viruses [10]. The first isolation dates back to 1968, when a group of virologists described the structure of a new group of viruses—isolated from humans, mice (mouse hepatitis virus), and avian species (avian infectious bronchitis)—and sent their conclusion to *Nature* [11]. They highlighted a characteristic common fringe of 200 Å long rounded to petal-like projections from the viral membrane, having the appearance of the “solar corona”, subsequently also identified as a “crown”, hence *Coronavirus* from the solar corona-like shape and the Latin word *corona* that means crown [10]. These projections constitute the typical “Spike” glycoproteins, which characterize all CoV membranes.

All CoVs belong to order Nidovirales, suborder Cornidovirineae, family Coronaviridae, subfamily Coronavirinae [12]. The members of this subfamily can be divided into four genera: Alphacoronavirus, Betacoronavirus, Gammacoronavirus, and Deltacoronavirus. Alpha- and Beta-coronaviruses affect mammals, Gammacoronaviruses cause diseases in avian species, whereas Deltacoronaviruses rarely infect mammals with a more specific tropism for birds [10,13]. Further division of genera into subgroups/subgenera is also described [12].

Not only do CoVs affect several species, causing a broad spectrum of disease, but some of them also have a zoonotic potential and the ability to jump from one reservoir species to a different species, including humans, usually through a bridging species. Regarding the two most relevant examples, the dromedary camel (*Camelus dromedarius*) has been identified as a bridging species of MERS-CoV that most likely spilled over from bats, whereas SARS-CoVs jumped to humans from civets (*Paguma larvata*) infected by maintenance bat hosts such as *Rhinolophus sinicus* and *R. ferrumequinum* [14,15]. SARS-CoV-2 has been analyzed throughout genome sequencing, showing 96.2% overall genome sequence identity with Bat CoV RaTG13, suggesting that Bat CoV and human SARS-CoV-2 might share a common ancestor [16]. As an example, one SARS-CoV-2-related coronavirus isolated from a Malayan pangolin showed 100%, 98.6%, 97.8%, and 90.7% amino acid identity with SARS-CoV-2 envelope (E), membrane (M), nucleocapsid (N), and spike (S) genes respectively, and therefore, whether SARS-CoV-2 has other reservoirs and/or intermediate hosts still remains a question to be addressed in the current scenario [17,18].

In light of the CoVs’ zoonotic risk and their ability to adapt to new species, it appears pivotal to understand the pathophysiology and mechanisms of tissue injury of known CoVs within the “One-Health” concept [12].

Even though there are still many doubts regarding the pathophysiological mechanisms in animals, this paper provides brief and general information on the CoV pathogenesis, together with a review of old and new CoV-associated animal diseases. Because the disease in bats is generally asymptomatic and very little information is available on associated lesions, these animals are not included in this review. The main aim of this review is to focus on gross and microscopic lesions associated to CoVs in different species, including pathogenesis when available.

## 2. Pathogenesis

### 2.1. Viral Life Cycle

**Scheme 1:**
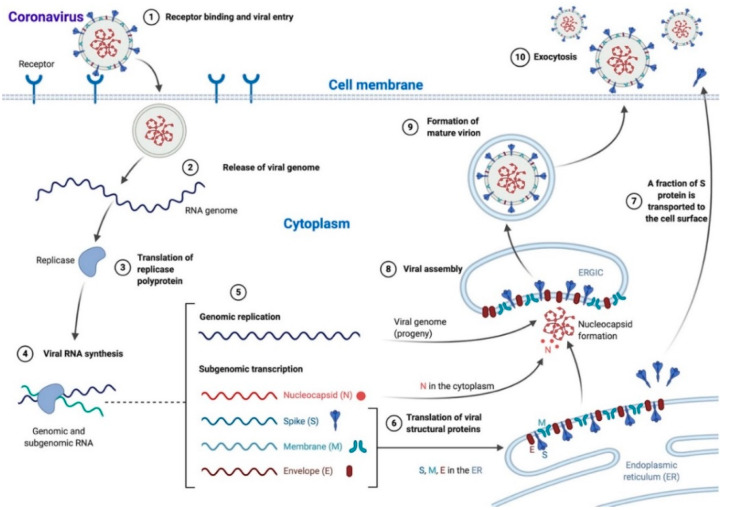
Coronavirus life cycle. Most Coronaviruses enter the cell after S protein–receptor interaction (1), the viral genome is released in the cytoplasm (2) and translated into the replicase polyprotein (3) to synthetize viral RNA (4). Genomic (progeny) and subgenomic RNAs are produced (5) and the latter are translated to structural and accessory proteins that can be inserted in the endoplasmic reticulum (ER; 6) and moved to the endoplasmic reticulum-Golgi intermediate compartment (ERGIC), while a fraction of S protein is transported directly to the cell surface where it mediates cell–cell fusion (7). In the ERGIC, the viral assembly and the encapsidation of genomic RNA by N protein take place, leading to nucleocapsid formation (8). Mature virions are then transported in smooth-walled vesicles (9) and released via exocytosis (10). N: nucleocapsid, S: spike, M: membrane, E: envelope. The scheme has been created with BioRender.com.

**Scheme 2:**
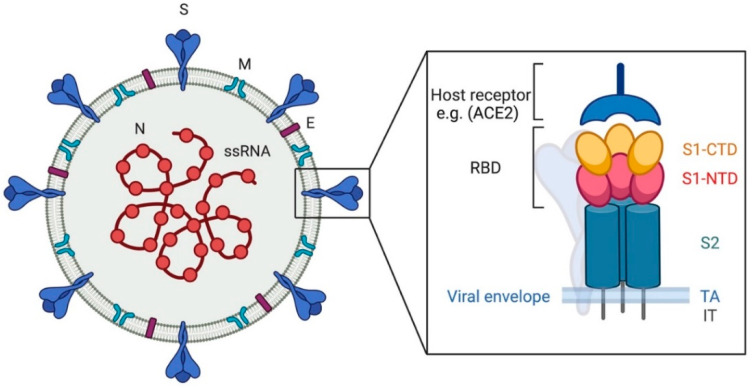
Coronavirus, viral particle. The image shows, on the left, a graphic representation of a viral particle. The black box shows the structure of the spike protein. N: nucleocapsid, S: spike, M: membrane, E: envelope. RBD: receptor binding domain, S1-CTD: subunit 1 C-terminal domain, S1-NTD: subunit 1 N-terminal domain, S2: subunit 2, TA: transmembrane anchor, IT: intracellular tail. The scheme has been created with BioRender.com.

The viral life cycle has common steps for all studied CoVs, both in humans and animals (Scheme 1). CoV infection begins with the interplay of the virion (Scheme 2) with host cells. Various specific host cell receptors mainly mediate viral entry strategies. These strategies and the receptors’ tissue distribution influence the viral tropism and pathogenicity [19,20]. Among some of the recognized CoV receptors there are the well-known angiotensin-converting enzyme 2 (ACE2) for SARS-CoV and SARS-CoV-2, the dipeptidyl peptidase 4 (DPP4, also known as CD26) for MERS [12], the aminopeptidase N—mainly used by *Alphadoronavirus*, and 5-N-acetyl-9-O-acetyl neuraminic acid (Neu5,9Ac2) and Carcinoembryonic antigen-related cell adhesion molecule 1 (biliary glycoprotein) (CEACAM1)—mainly for *Betacoronavirus* [19].

Viral attachment is predominantly mediated by the S protein–receptor interaction. Receptor binding causes major structural changes in the S protein (Scheme 2, see below), leading to viral fusion with the host cell membrane and access to the cytoplasm, where the viral genome is released [21] (Scheme 1). Depending on viral strain and on host cell type, this fusion process can occur directly at the cell surface or can be mediated by endocytosis [19]. Neurotropic CoV strains (i.e., mouse hepatitis virus, JHM strain) can mediate receptor-independent virus entry into host cells as a unique ability among viruses [10].

Once within the cytoplasm, the viral genome translates into the replicase polyprotein, which then uses the genome as a template for viral RNA synthesis. Viral RNA synthesis produces, through negative-strand intermediates, both genomic and subgenomic RNAs, the latter being translated into structural and accessory proteins [22] (Scheme 1). Viral structural S, E, and M proteins are inserted into the endoplasmic reticulum (ER) and then move to the endoplasmic reticulum-Golgi intermediate compartment (ERGIC) where viral assembly takes place (Scheme 1). Encapsidation of genomic RNA by N protein leads to nucleocapsid formation, followed by the production of mature virions through budding and association with ERGIC membranes containing viral structural proteins [22]. This process is mainly directed by M protein, which controls the interactions between structural proteins during virion assembly [23]. Mature virions are then transported to the plasma membrane in smooth-walled vesicles and released via exocytosis (Scheme 1). In some CoV strains, a fraction of S protein is not assembled into mature virions but gets transported to the cell surface (Scheme 1) where it mediates cell-to-cell fusion with the formation of multinucleated syncytial cells. As such, it facilitates the infection in adjacent cells without the need for extracellular viruses and the consequent escape of the host immune surveillance [10,22]. The site of virion release can differ between CoVs. For instance, transmissible gastroenteritis virus (TGEV) in swine is preferentially released at the infected host cells’ apical membrane, while the mouse hepatitis virus (MHV) favors the basolateral cellular surface. This difference in the release site can influence viral pathogenicity, as in this case, TGEV usually causes a localized enteric infection with an intraluminal release, while MHV can cause systemic disease [24].

### 2.2. Viral Spike Protein

The S protein plays a major role in CoV pathogenesis, host and tissue tropism, and host immune response. The CoV S protein is a large class I viral membrane fusion (transmembrane) glycoprotein composed of three segments: a large ectodomain, a transmembrane anchor, and an intracellular tail (Scheme 2). The ectodomain consists of a receptor-binding subunit S1 (N-terminal) and a membrane-fusion subunit S2 (C-terminal) [10,25]. S1 subunit, in turn, has two major subdomains, an N-terminal domain and a C-terminal domain: one or both these subdomains can function as a receptor-binding domain (RBD), binding sugars or recognizing protein receptors, respectively [10]. The S2 subunit comprises the fusion peptide and is responsible for membrane fusion [10,12,19].

After receptor binding, CoV S protein goes through major conformational changes. These changes are necessary for virus–cell fusion and entry. They consist of proteolytic processing of S protein itself by host proteases. Host proteases are, therefore, crucial for membrane fusion and entry. The most important source of these proteolytic enzymes is represented by the lysosomal proteases found in virtually all cell types. Additional tissue-specific availability of these enzymes can most likely influence tissue tropism of CoVs [10]. Similarly, the spillover potential of CoVs is also influenced both by the RBD–receptor interaction and by this proteolytic processing of S protein [12,26].

CoV S protein’s role is not only limited to viral fusion and entry. S protein is one of the major immunogens in CoVs and the main target of neutralizing antibodies in natural infections [26]. Moreover, S protein is also thought to have a key role in altering innate antiviral immune response through translational repression of mRNA transcripts, thus inhibiting interferon (IFN) and cytokine production, favoring viral infection and spreading [27]. However, several other immunogenic CoV proteins are investigated, particularly in human diseases, to understand deeper specific immune responses better and in order to develop efficacious vaccination campaigns.

### 2.3. Viral Mutation and Recombination

One of the striking features of CoVs is surely represented by their genetic plasticity. There is a high frequency of genetic changes for many CoVs, which forms the basis of their zoonotic potential [28]. For this, CoVs exploit two major mechanisms: mutation and recombination.

### 2.4. Mutation

Like in other single-stranded RNA viruses, genomic mutations do occur in CoVs [29] and are mainly due to their viral replicase, which does not possess good proofreading, but is efficient enough in maintaining large genomes without accumulating catastrophic errors and leading to progressive differentiation of viral progeny [28].

Mutational events can affect the CoV pathogenicity and host range. A striking example of how mutations influence tissue tropism and pathogenesis can be found in TGEV and porcine respiratory coronavirus (PRCoV). TGEV can infect both intestinal and respiratory cells, while PRCoV, an attenuated variant of TGEV, can only infect the respiratory tract, even if it binds to the same receptor as TGEV (porcine amino-peptidase N). This difference in tissue tropism might derive from the lack of hemagglutinating activity of PRCoV as a consequence of a deletion in the S1 domain compared to TGEV. Therefore, PRCoV is incapable of infecting intestinal cells, and its pathogenicity is consequently reduced [19]. Another example of mutational events that causes tissue tropism change can be found in the different pathogenicity between the widespread feline enteric coronavirus (FECV) and the lethal feline infectious peritonitis virus (FIPV). FIPV develops in individual cats persistently infected with FECV. Mutations in accessory and S genes could enable the virus to efficiently replicate in monocytes and macrophages, leading to the diffuse and lethal disease caused by FIPV, rather than the mild enteric form induced by the enterotropic FECV [30,31].

Mutations do not only influence CoV tissue tropism and pathogenicity but are also a key event for virus spillover. While the mutational events that have led to SARS-CoV-2 spillover are still not clear, the genetic rearrangements that caused SARS-CoV to host jump and outbreak have been extensively described. SARS-CoV is thought to have passed from bats, considered the natural reservoir for most animal CoVs, to humans using palm civets [32]. Both human and palm civet CoVs bind to the ACE2 receptor, but studies have shown that human SARS-CoV can bind both human and palm civet ACE2, while palm civet virus cannot bind human ACE2. This difference in receptor selectivity has been related to two-point mutations in the RBD of the human virus, showing its adaptation to the new host [19]. More recently, Korber and collaborators described an amino acid change in the SARS-CoV-2 spike protein caused by an A-to-G nucleotide mutation that led to a new variant, namely G614 [33]. The authors hypothesized that this new variant may have a fitness advantage over the original D614 form. Most importantly, they reported that the G614 variant was associated with potentially higher viral loads in COVID-19 patients, even though there was no significant association with increased disease severity [33].

### 2.5. Recombination

The marked tendency of CoVs to recombine with other CoVs (homologous recombination) and with RNAs of different viruses and other organisms (heterologous recombination) is related to their particular replicating machinery, as thoroughly described in a recent review [28]. Moreover, their exceptionally large RNA genome increases the probability of both mutational and recombination events. Genetic recombination has been extensively documented in both animal (i.e., MHV, TGEV, feline CoV, canine CoV) and human (i.e., OC43, NL63, HKU1, SARS-CoV, MERS-CoV) CoVs [29]. One of the best examples of genetic recombination in CoVs can be found in serotype II feline CoV (FCoV). This virus originates from the recombination event between FCoV and canine CoV (CCoV). Through this homologous recombination, serotype I FCoV acquires the CCoV *S* gene, including its neighboring regions, resulting in a change in the receptor-binding domain with critical biological consequences [31]. Serotype II FCoV can uses the feline aminopeptidase N (fAPN), a metalloprotease expressed in many host tissues, whereas it has been proven that serotype I FCoV uses different host cell receptors [31].

## 3. Coronavirus Diseases in Animals

**Scheme 3:**
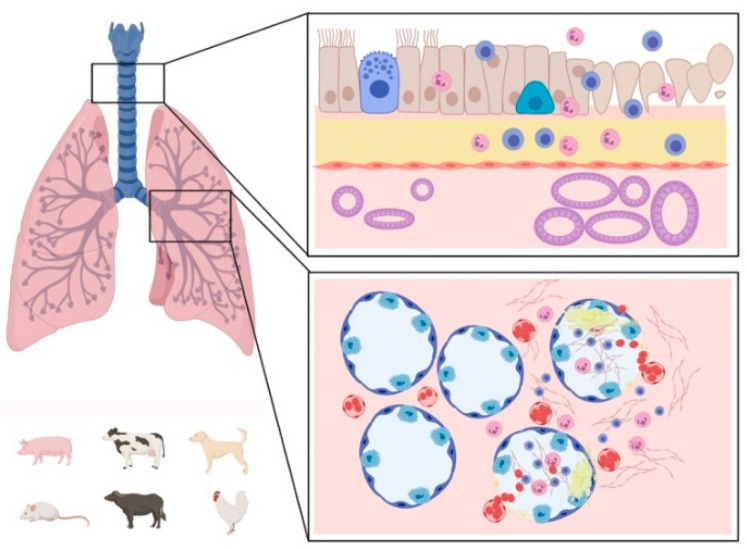
Graphic representation of the main respiratory lesions associated with Coronavirus infections in animals. Affected upper (top box) and lower (bottom box) respiratory tract: the healthy tissue is represented on the left side of the boxes and the lesions on the right side. The species mainly affected by respiratory lesions are represented on the lower left corner. Lesions of the upper airways are mainly characterized by mucosal inflammation consisting of neutrophilic (or heterophilic) and lymphocytic infiltrate, sloughing of epithelial cells, loss of cilia, epithelial cell hyperplasia, and hypertrophy of mucosal glands. Lesions in the lungs are mainly characterized by hemorrhages with neutrophilic (or heterophilic) and lymphocytic infiltrate, severe edema, epithelial cell detachment, and fibrinous exudate. The scheme has been created with BioRender.com.

**Scheme 4:**
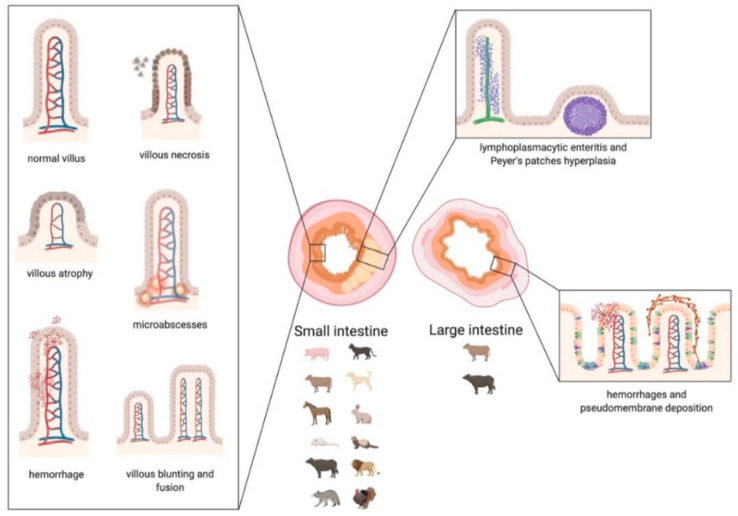
Graphic representation of the main lesions detected in small and large intestine associated with Coronavirus infections in animals. Small intestine: in the left box, the morphologic changes of the villi are represented. In the upper right box, the distribution of the infiltrate, affecting the lamina propria and the submucosa, is represented. Large intestine: in the lower right box, the lesions affecting the mucosa of the colon are represented. The main categories of affected species that suffer damage mainly to the small or large intestine are indicated below the corresponding image. The scheme has been created with BioRender.com.

**Scheme 5:**
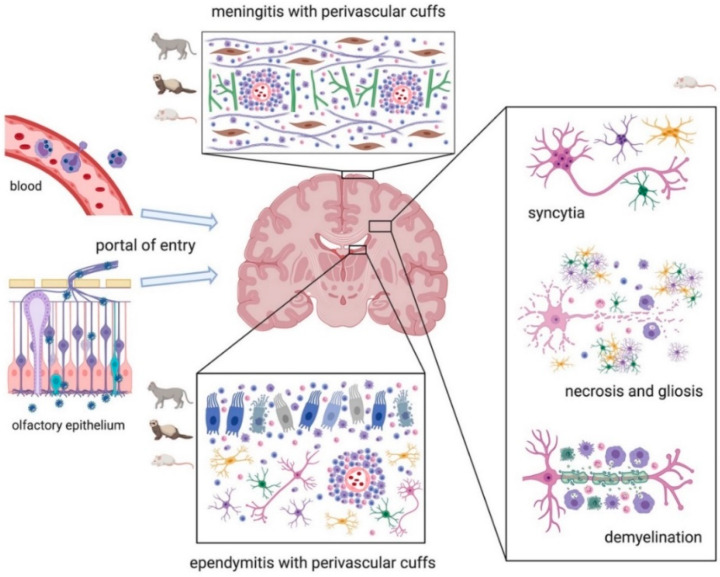
Graphic representation of the main brain lesions associated with Coronavirus infections in animals. Typically, the virus disseminates to the brain either via the blood within monocytes or along the olfactory nerve through the olfactory epithelium (left of the image). Main lesions affecting cats, ferrets, and mice are represented by meningitis and ependymitis with perivascular cuffs. Lesions in mice are also characterized by syncytia of neurons, astrocytes, microglia, and oligodendrocytes. Necrosis and gliosis are common features in laboratory animals. Chronic infections in mice can result in demyelination. The scheme has been created with BioRender.com.

The major lesions and affected organs in CoV-associated diseases in animals are summarized in Table S1 and the main tissues affected are schematically represented in Scheme 3, Scheme 4 and Scheme 5. A brief description of disease classification, distribution, and mainly gross and histological findings per animal species group follows.

### 3.1. CoVs in Pets (Dogs and Cats)

CCoV is an important pathogen of dogs diffused worldwide, especially in kennels and animal shelters [34,35]. In dogs, three subtypes of CCoVs have been identified [35,36]. CCoV type I and II belong to *Alphacoronavirus*, as does FCoV. Type I CCoV shows more genetic similarity to FCoV type I, while CCoV type II, which can be divided into classical subtype II-a and recombinant II-b, is genetically closer to FCoV type II [34,35]. The third CCoV, Canine Respiratory Coronavirus (CRCoV), belongs to *Betacoronavirus*, like Bovine Coronavirus (BCoV), to which it is genetically close (96% amino acid identity with BCoV in the variable S protein) [34,36,37].

CCoV type I and II cause mild asymptomatic enteritis, usually self-limiting, except for highly pathogenic variants of CCoV type II [38].

FCoV, distributed worldwide like CCoV, has two distinct serotypes, type I FCoV and type II FCoV. Type II FCoV results from a double recombination between type I FCoV and CCoV type II [31,38,39]. FCoVs occur as two different pathotypes: FECV, the enteric highly contagious ubiquitous form, and FIPV, the most virulent form that affects about 5% to 12% of FCoV-infected cats, probably after specific mutations in the viral genome of infected cats [31,39,40]. Both FCoV type I and II can cause FIP, even if studies confirmed that FCoV type I is more frequent [39].

While for many of these coronaviruses the pathogenesis is not completely clear, numerous studies investigated how FCoVs can cause disease. FECV has tropism mainly for the apical epithelium of intestinal villi from the small intestine to the cecum, even if it can also infect monocytes. Typically, FIPV infects and then replicates in cells of monocyte/macrophage lineage. After infection, monocytes are activated and express cytokines that facilitate their interaction with activated endothelial cells in small- and medium-sized veins. It has also been suggested that the increased expression of enzymes such as metalloproteinase-9 by activated monocytes contributes to endothelial barrier dysfunction and subsequent extravasation of monocytes, with typical perivascular and vascular damage [31,39,41].

FECV causes infections usually restricted to the gastroenteric tract, characterized by high morbidity and low mortality, similarly to CCoVs [31,39,40]. There are often no clinical signs in old cats, but in kittens, not protected by maternal antibodies, FECV can cause severe catarrhal to hemorrhagic enteritis [39].

Grossly, infection of monocytes and macrophages in FIP manifests as typical lesions consistent with fibrinous and granulomatous serositis, with effusions and a typical systemic infection with pyogranulomas in several organs. FIP’s two main forms are: effusive and non-effusive (dry or granulomatous), even if postmortem examination often identifies mixed forms [31,39,40]. In most cases, the peritoneum is often involved, associated with abdominal effusions and sometimes effusions in the thorax. The typical inflammatory exudate of FIP is more frequently encountered in kidneys, omentum, hepatic and splenic capsule, and may extend into the parenchyma, but other organs can also be affected [40]. Differently from other CoVs of pets, FIP (dry form) can be restricted to a single organ [40], or it can reach atypical sites for coronavirus infections causing, for example, orchitis [42], priapism [43], and lesions in the skin [44] and the myocardium [45]. Eyes, kidneys, and brain can also be affected during FIP infection [40,46].

Rarely, CCoV can cause fatal and systemic diseases, and some outbreaks caused by a pantropic II-a CCoV have been described in recent years [35]. Cases of infection with this pantropic II-a CCoV strain CB/05 were mainly characterized by hemorrhagic enteritis and abundant serosanguineous fluid in the abdominal cavity. Additionally, the lungs showed multiple areas of consolidation and peripheral emphysema. Hemorrhages were also evident in the liver, spleen, kidneys, and often associated with enlargement of mediastinal and mesenteric lymph nodes [38,47]. More recently, a new strain of CCoV type II-a has been identified (strain 450/07), after the death of a 60-day-old miniature pinscher with similar hemorrhagic enteritis, fibrinous pneumonia, and multiorgan involvement [37]. Previously, in 2005, Evermann and colleagues had already reported two cases of fatal CCoV in two dogs. In both cases, the primary lesions were intestinal, with hemorrhagic enteritis, and in one case also associated with mild interstitial pneumonia [48]

CRCoV is a *Betacoronavirus*, but it is usually related only to mild respiratory clinical signs, and it is one of the most common pathogens of the canine infectious respiratory disease (CIRD), often associated with other pathogens like Canine Parainfluenza Virus and *Bordetella bronchiseptica* [49,50,51]. CRCoV rarely causes severe infections as a single agent.

**Figure 1:**
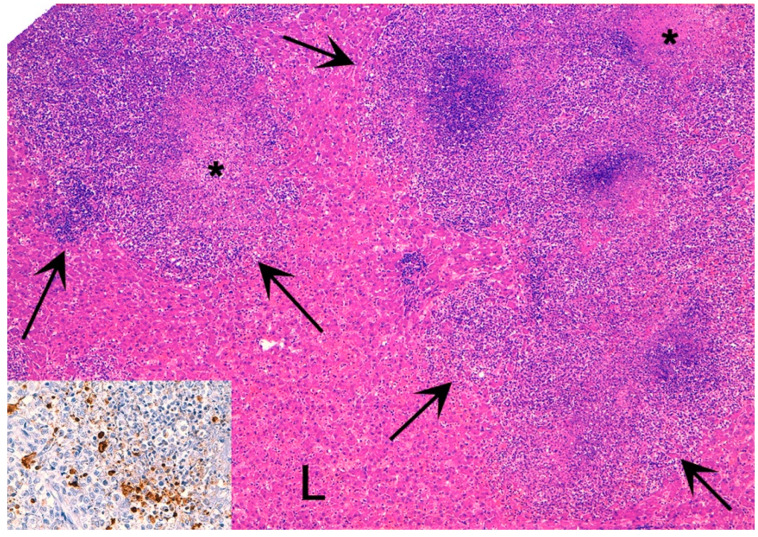
Feline infection peritonitis (FIP), liver (L), cat. Hepatic parenchyma is effaced by a multifocal to coalescent mixed inflammatory infiltrate (arrows) with central necrosis (asterisks). Hematoxylin eosin stain. Inset: Immunohistochemistry shows intense brown cytoplasmic immunolabelling for feline CoV antigen within macrophages.

On histology, the most well-characterized lesions are those of FIP that causes typical granulomatous to necrotizing periphlebitis and phlebitis with perivascular B-cell and plasma cell infiltrates [39]. Phlebitis is usually limited to small- and medium-sized veins in the leptomeninges, renal cortex (stellate veins), eyes (venules in iris, choroid, and retina), and, less frequently, in lungs and liver, it is mainly mediated by virus-infected monocytes/macrophages (with the presence of few T-cells and neutrophils) intended to progress into granulomatous lesions (Figure 1) [39,41]. Necrosis in vessel walls and sporadic smooth muscle hyperplasia are mainly caused by perivascular macrophages [40], while monocytes rarely infiltrate the wall and usually adhere to endothelial cells [41]. B-cells and plasma cells tend to replace macrophages progressively into the granulomatous infiltrate [39]. Lymphoid tissues typically show T- and B-cells’ depletion, while in the splenic red pulp, the number of macrophages may increase [52].

Histologic lesions of the pantropic form of CCoVs show some similarities with FIP. In CCoV fatal outbreaks, lungs present a fibrinopurulent infiltrate extending from alveoli into bronchioles and bronchi, macrophages with erythrophagocytosis in the alveoli, perivascular serous-fibrinous edema, and mural fibrinoid vascular necrosis, with vascular walls infiltrated by monocytes and neutrophils (the latter are rare in FIP). The spleen shows multifocal hyperemia and diffuse severe lymphoid depletion, also evident in thymus and lymph nodes, similar to FIP [47,48]. The small intestine shows chronic lymphoplasmacytic inflammation with mild fibrosis, and crypts’ necrosis associated to necrosis of villi, to a lesser extent [47,48]. Areas of necrosis have also been reported in the bronchial and bronchiolar epithelium, and in the liver and kidney [47].

By means of immunohistochemistry (IHC), pantropic CCoV has been detected within the cytoplasm of macrophages in the lungs around bronchial/bronchiolar epithelium and within alveolar septa and lumina, in arteriolar walls, within Kupffer cells, within perivascular cells in the liver, in gut and lymph nodes, and rarely, in perivascular areas of spleen and kidney [38,47].

The less aggressive FECV usually does not cause marked lesions. However, in kittens with more severe infections, there can be villous atrophy, fusion of adjacent villi, and sloughing of the mucosal epithelium at the level of duodenum, jejunum, and ileum. Through IHC, FECV has been detected in villous epithelial cells [39,53,54]. Similar to what happens in immunocompetent cats, histologic lesions of CCoV infections are not frequently reported because of the mild nature of this infection. Lesions include rare atrophy of intestinal villi, lined by attenuated low-cuboidal to squamous epithelial cells, and lymphoid depletion of Peyer’s patches. With the use of IHC, similarly to FECV, the viral antigen has been detected in the cytoplasm of villus epithelial cells [55].

CRCoV histologic lesions have not been described in naturally infected dogs, but, in 2012, a study reported lesions in experimentally infected puppies that received intranasal virus inoculations. The authors detected inflammation in the trachea and nares with loss of mucosal cilia. Only mild abnormalities were present in the lungs, consisting of lymphoid aggregates adjacent to airways or blood vessels. IHC revealed the presence of CRCoV in the tracheal epithelium and bronchioles of infected dogs [51]. These data are consistent with histopathological lesions usually observed in the early stages of CIRD (mild to moderate tracheobronchitis), which may support the hypothesis that CRCoV alters the mucociliary clearance in the upper airways, predisposing dogs to secondary infections [56].

Considering comparative aspects with human CoVs, Tilocca and collaborators analyzed the sequence of epitopes of SARS-CoV-2 spike proteins and found a high percentage of homology with other taxonomically related CoVs, such as BCoV, Human Enteric Coronavirus, and CRCoV. In detail, CRCoV showed the highest homology with one of the four SARS-CoV-2 analyzed epitopes [57]. CRCoV has also been proposed as a model for COVID-19 by Priestnall, who explains that CRCoV, even if mainly present in respiratory tissues, has been detected in the spleen, mesenteric lymph nodes, and colon, which may suggest a potential dual tropism [58]. Similarly, there is evidence that SARS-CoV-2 can infect the human gastrointestinal system [58]. As a comparative feature, it is also essential to mention the so-called “cytokines storm” or cytokine release syndrome (CRS). In a previous study on canine tracheal explants, an increase of interleukin-6 (IL-6) mRNA level has been observed 96 h after CRCoV inoculation, and IL-6, in particular, has been suggested as critical molecule for CRS in humans with most severe COVID-19 disease [58]. Another exciting hypothesis that links CCoV and SARS-CoV-2 is related to *5′-C-phosphate-G-3′* (CpG) dinucleotides deficiency, associated with the Zinc-finger-antiviral-protein (ZAP). Many viruses have developed CpG deficiency, depending on the ZAP activity of their target tissues. SARS-CoV-2 has the greatest CpG deficiency among all *Betacoronaviruses,* and suggests that SARS-CoV-2 may have evolved in a host with high ZAP expression [58]. The greatest CpG deficiency among all viral genomes has been found in pantropic CCoV (an *Alphacoronavirus* and not a *Betacoronavirus* like SARS-CoV-2 or CRCoV), suggesting that dogs infected with CCoV may have created a strongly selective anti-CpG environment and may be involved in SARS-CoV-2 origin [59].

Recently, some studies focused on the role of domestic animals in COVID-19 outbreaks, and few experimental inoculations suggest that cats are susceptible to SARS-CoV-2. One study showed that cats with SARS-CoV-2 can transmit the virus to other cats, since, after cohousing with SARS-CoV-2-positive subjects, all nasal swabs of SARS-COV-2-free cats resulted positive [60]. In an in-depth study, following intranasal inoculation in cats, the infective virus was detected in the nasal turbinates, soft palate, trachea, tonsils, and lungs, and viral RNA was found in the small intestine and feces of inoculated cats. Moreover, cats exposed to SARS-CoV-2, which were in the same household as non-treated inoculated cats, presented viral RNA in the upper airways. In the same study, in younger cats, SARS-CoV-2 caused severe lesions in the nasal and tracheal mucosal epithelium and in the lungs, and all the inoculated cats seroconverted. Dogs were also tested for the presence of the infection in the same study, but not all swabs of the inoculated dogs resulted positive. Viral RNA was not found in any organ, and not all inoculated dogs seroconverted. These results suggest that SARS-CoV-2 can replicate efficiently in cats and that it can be transmitted among cats through the respiratory route, while dogs seem to have lower susceptibility [61].

### 3.2. CoVs in Ferrets

Ferret enteric CoV (FRECV, MSU-2 strain, and No22 strain), belonging to the *Alphacoronavirus* genus, *Minacovirus* subgenus, was identified in feces of domestic ferrets clinically diagnosed with epizootic catarrhal enteritis (ECE). The microscopic lesions observed in affected ferrets were consistent with those described in the intestinal CoV infections in other species [62,63]. The mucosa of the affected portion of the small intestine is usually highly hyperemic and thin. A diffuse lymphocytic enteritis, with villus atrophy, fusion, and blunting, and vacuolar degeneration and necrosis of the apical epithelium, is often detected. Transmission Electron Microscopy (TEM) studies revealed CoV-like particles in the feces and in the affected jejunal enterocytes of ferrets with ECE. FRECV, MUS-2 strain RNA, and antigen were detected in the cytoplasm of enterocytes at the villi tips in the jejunum of affected ferrets. FRECV RNA or antigen was not found in the large intestine, lymph nodes, spleen, esophagus, stomach, and parotid salivary glands [64].

Ferret systemic coronavirosis is an emerging fatal disease of ferrets caused by ferret systemic coronavirus (FRSCV), belonging to *Alphacoronavirus*, Minacovirus subgenus, which shares clinical-pathological characteristics with the dry form of FIP in cats. FRSCV was described as causing multisystemic granulomatous lesions [63,64,65,66,67]. Serosa and gastro-intestinal tract are usually affected, but many other visceral organs, including the brain, are also involved. Rare multinucleated giant cells have been described. Granulomatous inflammation is often localized around vessels and frequently involves the adventitia, with inflammatory cells migrating into the medial tunics of small veins and venules [68,69]. Clear signs of vasculitis were not described, but this might represent an early event progressively obscured by granulomas development, remarkably similar to FIP, despite being slower in progression. FRSCV viral antigen was detected in the cytoplasm of macrophages.

Similar to cats, ferrets have been studied to clarify their potential epidemiological role in COVID-19 outbreaks. In Reference [61], after intranasal inoculation of SARS-CoV-2 human isolates, viral RNA was detected in the nasal turbinates, soft palate, tonsils, and, although in lower copy numbers, in some rectal swabs. Infectious virus was detected only from nasal swabs. Antibodies against SARS-CoV-2 were detected in all of the ferrets by an enzyme-linked immunosorbent assay (ELISA) and a neutralization assay. However, other experimental inoculation studies on ferrets [70,71] show that viral RNA may also be present in lungs, muscles, cerebrum, cerebellum, skin, trachea, lung lymph nodes, and intestine. Furthermore, the virus could also be cultivated from the lungs. Richard and collaborators showed that SARS-CoV-2 is transmitted efficiently via direct contact and via the air (via respiratory droplets and/or aerosols) between previously inoculated and healthy ferrets [72]. SARS-CoV-2-infected ferrets shed virus in nasal washes, saliva, urine, and feces up to 8 days post-infection [71]. Although no gross lesions were reported in any of the studies, inoculated and euthanized ferrets showed lung histopathological lesions consistent with severe lymphoplasmacytic perivasculitis and vasculitis, with increased numbers of type II pneumocytes, macrophages, and neutrophils in the alveolar septa and alveolar lumen, and a mild peribronchitis [61].

### 3.3. CoVs in Bovines

BCoV is an ancestor of many other coronaviruses affecting other species, such as humans (HCoV-OC43), swine (Porcine Hemagglutinating Encephalomyelitis virus—PHEV), equids (ECoV), and dogs (CRCoV) [28]. It is also able to infect mice and several ruminant species [23].

BCoV has a tropism for the intestinal tract, nasal passages, proximal trachea, and lungs [73], and therefore is associated either with a respiratory syndrome (isolated from cases of both calf pneumonia and shipping fever, in association with other pathogens) or an enteric syndrome, which includes diarrhea in calves and winter dysentery in adults [74,75]. Based on this, BCoVs includes BeCoVs (bovine enteric or enteropathogenic coronaviruses) [76] which can cause BeCoV-induced calf diarrhea (BeCoV-CD) and winter dysentery (BeCoV-WD) [73], and Bovine respiratory CoVs, which can cause calf pneumonia and contribute to shipping fever [77].

All BCoV isolates examined to date, regardless of clinical origin, belong to a single serotype [77]. Macroscopic findings associated to the respiratory form were first reported in 1984 as pneumonia, pericarditis, pleuritis, and septicemia [78]. At necropsy, pathologic changes are visible in approximately 30% of the animals [79]. They can range from multifocal to coalescing areas of consolidation in the ventral portion of the lungs to subacute exudative and necrotizing lobar pneumonia involving 50–80% of the pulmonary parenchyma when associated to secondary bacterial infections [80]. Adhesions are rarely present, but fibrin tags on thoracic pleura occurred in 40% of animals with pulmonary lesions. The trachea of affected animals usually shows petechiae on the mucosal surfaces and variable quantities of mucopurulent discharge, which can also be present in the bronchial lumina [79]. In neonatal colostrum-deprived calves, lesions reported comprise interstitial pneumonia, emphysema, pulmonary congestion, and hemorrhage, with interstitial edema [79].

With the enteric form, the spiral colon, and, to a lesser extent, rectum are the sites of choice to detect BeCoV at postmortem examination. These organs show the main gross findings, and the virus persists in these specific locations for the longest time after oral infection [73,81,82]. Boileau and collaborators deeply revised the literature on BCoV infections and observed that both BeCoV-CD and BeCoV-WD are associated with similar intestinal lesions, with muco-hemorrhagic enterocolitis being the main gross finding [73,82,83]. Mesenteric and ileal lymph nodes can be enlarged and congested [84]. The spiral colon is edematous and fluid-filled [82] with petechiae, ulcers, and diphtheritic membranes that can show a segmental distribution [82,83,84,85].

In 2019, Ellis published a complete review of respiratory histological lesions, describing tracheal petechiae with mucopurulent material and bronchointerstitial pneumonia with intra-bronchiolar syncytial cells as typical lesions [86]. Necrosis of epithelial cells in the nasal turbinates, trachea, bronchioles, and pulmonary parenchyma, with associated interstitial pneumonia and hyperplasia of type II pneumocytes, has been described in detail [79,80,87]. In the BCoV infection, the syncytia are confined to the bronchioles, while syncytia associated with bovine respiratory syncytial virus (BSRV) may be found in both airways and alveoli [88]. Interestingly, syncytial cells have also been observed in humans infected with severe acute respiratory syndrome coronavirus (SARS-CoV) [79].

In experimentally infected calves, the lesions appear to progress from the duodenum to jejunum, ileum, cecum, colon, and rectum [87]. Microscopically, the mesenteric lymph nodes show moderate depletion of cortical follicles or even necrosis of the follicles [84]. The mesenteric vessels’ tunica media may expand because of abundant edema, fibrin, neutrophils, lymphocytes, histiocytes, and plasma cells (vasculitis) [82].

The small intestine shows sloughed villi, villous atrophy, widespread villous fusion, increased crypt depth, crypt micro-abscesses (similar to equine CoV), hyperplasia of crypt lining cells, and an engorged or hemorrhagic lamina propria infiltrated by mononuclear cells [84,87]. In the large intestine, the most common lesion is acute hemorrhagic colitis. Additionally, there are reports of mucosal ulcers with fibrin deposition, necrosis of intestinal crypts, and atrophy of the colonic glands [83,84,87]. The submucosal layer can be expanded by perivascular infiltrates of neutrophils, macrophages, lymphocytes, and plasma cells, extending into the mucosa [82,83].

Interestingly, among the four human CoVs responsible for mild respiratory diseases, two of them (HCoV-OC43 and HKU1) likely originated from rodents, and HCoV-OC43 spilled in bovines before infecting humans [12].

### 3.4. CoVs in Sheep and Goats

Bovine-like CoVs have been identified worldwide as a cause of enteritis and neonatal mortality in domestic sheep. A nationwide screening for BCoV-specific antibodies in Swedish sheep has demonstrated that 19% of all sera samples were positive, but no pathologic lesions were found [89]. In goats, only one study detected CoVs in the feces of one out of 19 (3.3%) kids with neonatal enteritis in Turkey, with viral positivity in the crypt epithelium of the small intestine and the submucosal macrophages [89]. Kandeil and colleagues provided the first evidence of MERS-CoV infection in sheep farmed in close contact with camelids [90]. They also found positive sheep, goats, and donkeys in Egypt, and a positive sheep and goat in nomadic herds in Senegal, all living in close contact with camelids, although necropsies were not performed.

### 3.5. CoVs in Equids

Equine CoV (ECoV) is a *Betacoronavirus* like BCoV, of which it is a descendant [28]. As such, it is essential to note that IHC performed using anti-BCoV antibodies cross-reacts with ECoV antigens, and vaccination against BCoV also protects horses against ECoV [91]. Also, there is a close antigenic relationship of ECoV with BCoV and the PHEV of pigs [92].

ECoV infections in adults and foals have been reported in the USA [92,93,94,95,96], Japan [97,98,99], and Europe [100].

ECoV can be fatal, with colic, fever, lethargy, inappetence, tachycardia, and signs of encephalopathy apparently related to hyperammonemia [93,95,96,101]. Similar symptoms were also found in a donkey [96]. However, up to 33% of infected horses have been reported to be asymptomatic by some authors [95].

A review from Pusterla and collaborators indicates that the most frequent clinical signs are represented by anorexia (97%), lethargy (88%), and fever (83%), with a clinical onset prior to gastro-intestinal signs, which are present in 10% of affected horses [102]. Neurological signs were instead present in only 3% of infected horses in the same study.

The infection begins in the proximal small intestine, where ECoV causes blunting and atrophy of villi, leading to malabsorption and maldigestion, and then spreads to colonic crypt cells [103].

In their review, Pusterla and colleagues suggest that hyperammonemia may be caused by the rupture of the gastro-intestinal barrier, leading to an increase in production or absorption of ammonia in the gastro-intestinal tract. Alternatively, microbiome imbalance might also induce changes in ammonia production [102].

Several organs can be affected during ECoV fatal infection, but the main gross lesions occur in the gastro-intestinal tract as diffuse fibrinous enteritis with multifocal pseudomembranes and the possible multifocal involvement of the large intestine [96]. Ascites and submucosal edema were observed primarily in foals [101]. Additionally, diffuse marked pulmonary congestion and edema were also present in some cases [96].

Histology confirms the diffuse necrotizing enteritis [95]. Jejunum and ileum are particularly affected with attenuation of villi, loss of lining epithelium, crypt micro-abscesses, fibrin, and pseudomembrane deposition, often with secondary bacterial colonization, and hemorrhages. The lamina propria and the submucosa are infiltrated by histiocytes, lymphocytes, neutrophils, and eosinophils with fibrin thrombi, which can be appreciated on postcapillary submucosal venules (microthrombosis). Crypt enterocytes may contain single 1.5 to 3 µm diameter, irregularly round intracytoplasmic eosinophilic inclusion bodies within clear vacuoles up to 4 µm in diameter. The ileum shows lymphocytolysis of Peyer’s patches [96]. The necrotizing nature of the lesions shows higher similarity to BCoV than other Coronaviruses, such as *Deltacoronavirus*, which may be more prone to cause atrophy [96]. Interestingly, Alzheimer Type II astrocytes in the cerebral cortex have been described in animals with hyperammonemia [95,96].

### 3.6. CoVs in Swine

Pigs can be infected by 6 CoVs, including four *Alphacoronaviruses*, one *Betacoronavirus*, and one *Deltacoronavirus*. Transmissible gastroenteritis virus (TGEV), Porcine Epidemic Diarrhea Virus (PEDV), Porcine *Deltacoronavirus* (PDCoV), and Swine Acute Diarrhea Syndrome Coronavirus (SADS-CoV) are linked to gastro-intestinal infections, PHEV (*Betacoronavirus*) causes both neurological and gastro-intestinal infections, and PRCoV is associated with respiratory infections [28].

TGEV, one of the first isolated CoVs from diseased domestic animals [104], replicates in porcine respiratory tract but does not induce primary respiratory disease [105,106,107].

Necropsies of naturally infected pigs showed an extreme thinning of the small intestine as the most remarkable lesion.

Classical signs are attributable to a small intestine disease (jejunum and ileum) and consist of mild–moderate to marked villous atrophy (depending on the stage of progression) [108], hyperplasia of crypts in jejunum and ileum, with more severe lesions in younger pigs (3 days old vs. 21 days old) [104], and subsequent regeneration of atrophic villi. The main affected tissue is represented by the epithelium of villi [109], but crypts can also be affected by hyperplasia [108].

PEDV is clinically and histologically indistinguishable from TGEV. However, these two *Alphacoronaviruses* are antigenically distinct and do not demonstrate serological cross-reactivity [110]. Both experimentally and naturally infected pigs presented yellow feces at postmortem examination, the stomachs contained little milk curd, and the small and large intestines were empty or distended by watery contents with undigested milk. The intestinal walls were transparent and thin, mainly affecting the small intestine, with different degrees of severity and extension from the duodenum to the colon [111,112].

Lesions are mainly distributed amongst duodenum and proximal and mid-jejunum [113]. The diagnosis reported by Jung and colleagues is of an acute diffuse severe atrophic jejunitis [112]. The enterocytes show necrosis and atrophy of villi [101,111,113,114,115]. Attenuation, swelling, flattening, karyomegaly, and cytoplasmic vacuolation of superficial enterocytes are commonly described [113,116,117]. The mean jejunal villous height to crypt depth ratio (VH:CD) decreases as the disease progresses due to the ongoing necrotizing process [111,112]. Similarly, the VH:CD ratio is half the control pigs’ ratio due to crypt hyperplasia [113]. Syncytial enterocytes were detected only by Stevenson and collaborators [111]. As previously stated, the lesions are confined to the duodenum, proximal, and mid-jejunum [113], except for subepithelial edema in the cecum and colon [112]. Interestingly, Park and collaborators described IHC immunopositivity in pulmonary tissues despite the absence of histological lesions throughout the respiratory tract [117]. Microminipigs, genetically similar to conventional pigs, were inoculated with PEDV and showed very similar lesions to their commercial counterparts [118]. They exhibited thinning of the intestinal wall in the duodenum, jejunum, and ileum at necropsy, and severe villus atrophy was confirmed histologically. The villous height to crypt depth ratio in the small intestine was markedly decreased, while the large intestine showed no pathological changes.

PDCoV lesions are similar to those caused by PEDV, but milder [116]. Interestingly, Ma and collaborators report a close relationship of American PDCoV with Hong Kong and South Korean PDCoV strains and CoVs of Asian leopard cats and Chinese ferret-badgers [119]. Notably, PDCoV is the only *Deltacoronavirus*, which spreads among avian species, that is able to infect domestic mammals.

The small intestine, cecum, and colon of pigs that were experimentally inoculated [120] appeared thin-walled and gas-filled in most of the pigs, with a yellow, soft to watery content, in the first week post-infection. The stomach usually contained curdled milk, and all the other internal organs presented no macroscopically visible lesions. The authors defined these lesions as “PEDV-like” [120].

The common diagnosis is a severe subacute and diffuse atrophic enteritis targeting the jejunum and ileum [121]. Mild vacuolation of superficial epithelial cells in the cecum and colon has also been observed by Jung and collaborators [120]. Multifocal to diffuse/mild to severe villous atrophy and villous attenuation and enterocytes loss, with necrosis and sloughing of degenerate enterocytes into the lumen are observed, and villus-to-crypt ratio is decreased to 2:1–3:1, similar to PEDV (significantly decreased average villus heights, increased average crypt depths, and lower average villus/crypt ratios in the middle and distal jejunum and ileum) [116,119,120,121,122]. Epithelial syncytia were rarely reported in the gastric glandular mucosa, duodenum, jejunum, and ileum [116,119,121,122]. Only Ma and collaborators reported lesions in the lung as mild multifocal non-suppurative interstitial pneumonia, however without convincing histologic evidence of epithelial cell necrosis or syncytia [119,121,122].

SADS-CoV appeared only in 2015, and only a little information is available [123]. The infection is quite similar in pathogenesis and lesions to PEDV and PDCoV [124].

Viral replication occurs quickly and mainly in the villous epithelial cells of the small intestine, resulting in marked villous atrophy due to necrosis [123,124]. No pathological changes in the cecum and colon have been described [124]. In in vitro studies, syncytia formation was observed [125].

Interestingly, recent studies demonstrated that SADS-CoV isolates from severe outbreaks in piglets were identical to bat CoV HKU2, suggesting a bat origin [123]. Receptor analysis demonstrated that none of the well-known CoV receptors (ACE2, DPP4, aminopeptidase N), relevant for human CoV diseases, are essential for SADS-CoV entry. Up to now, this appears as the first example of spillover from bats of a domestic animal CoV, causing severe disease.

PHEV has been recently thoroughly reviewed by Mora-Diaz and collaborators [126]. It is the only known neurotropic coronavirus in pigs and produces vomiting and wasting disease (VWD). PHEV can spread from the primary sites of replication through the peripheral nervous system to the central nervous system (CNS). Primary viral replication in the nasal mucosa and tonsils allows the virus to spread to the trigeminal ganglion and brainstem trigeminal sensory nucleus. Viral spreading through the vagal nerve also allows the virus to infect the vagal sensory ganglion and brainstem vagal sensory nucleus. The virus can also spread peripherally from the intestinal myenteric plexuses to the spinal cord’s local sensory ganglia. After peripheral viral spreading, the virus infects well-defined nuclei of the medulla oblongata, progressing to the brainstem, spinal cord, and occasionally cerebrum and cerebellum. Immunofluorescence staining in the brain revealed that the infection is always restricted to neurons’ perikaryon and processes. Vomiting is induced by viral replication in the vagal sensory ganglion (ganglion distale vagi) or by impulses of the vomiting center, induced by vagal ganglia-infected neurons. It has been suggested that virus-induced lesions in the stomach’s myenteric plexus may contribute to gastric stasis and delayed stomach emptying. PHEV replicates only in the cytoplasm of sensory neurons [127].

At postmortem examination, infected pigs do not show relevant gross findings, except for cachexia, a dilated stomach containing abundant ingesta (non-digested milk), and distension of the abdomen in some chronically affected piglets [126]. Lesions affecting the brain comprise non-suppurative encephalomyelitis, with lymphoplasmacytic perivascular cuffing, mononuclear cell infiltration in the gray matter of the cerebrum, neuronal degeneration, satellitosis, and gliosis affecting the mesencephalon, pons, medulla oblongata, horns of the proximal spinal cord, and trigeminal ganglia (ganglioneuritis is also described). Piglets showing VWD signs can present gastric lesions, mainly in the pyloric gland area, such as degeneration of the plexuses (submucosal and myenteric) and lymphoplasmacytic perivascular cuffing, present in 15–85% of affected animals. Many studies failed to detect microscopic lesions in the tonsils and respiratory tract despite being positive for the virus [128]. Syncytia were detected only in vitro [126].

PRCoV is a derivative of TGEV and can be asymptomatic or subclinical, without diarrhea [129]. After a respiratory replication, the viremia follows, and with the ingestion of the virus, there is a mild replication in few eneterocytes in the small intestine (resembling TGEV). PRCoV infects the upper respiratory tract, trachea, tonsils, or lungs, with limited intestinal replication, but the asymptomatic or subclinical form occurs most frequently [130].

Cox and colleagues performed an experimental infection through aerosol in pigs. At necropsy, pneumonia was present in about 78% of the inoculated animals. Macroscopic alterations in other organs were not detectable [129]. Microscopic lesions reported in the paper by Cox showed interstitial pneumonia associated with capillary congestion. The pneumonia became more severe as the disease progressed. Moreover, after four days of infection, there was focal degeneration of the alveolar interstitium in the apical and cardiac lobes [129].

### 3.7. CoVs in Wild Animals

There are limited studies regarding CoV infections in wild mammals. In wild populations, several factors might limit CoV infections, such as low animal density, limited interspecies transmission, absence of insect vectors, high lability of the virus outside the host, and restricted host range due to the lack of specific viral receptors. This is generally reflected in the low seroprevalence of wild animals, such as canids to CCoVs (1.7%), felids to FCoVs (2%), and various bovids to BCoVs (range, 6.6–13.3%) [131,132,133,134].

### 3.8. Wild Felids

In wild felids, FCoV infection can cause a variety of CoV-associated diseases. Cheetahs *(Acinonyx jubatus)* diagnosed with FIP (*Alphacoronavirus*) share many common features with domestic cats. Cheetahs’ exposure to FCoV varies according to the habitat and may reflect the contact with domestic cats or dietary exposure to cross-reacting coronaviruses of cheetahs’ prey [134,135]. Because most cheetahs are highly inbred, the outbreaks in this species might be associated with a lack of resistance to the infection in the entire population [136,137]. FIP in cheetahs may be due to inherent increased susceptibility, impaired host response, environmental factors leading to high host density, and reporting bias toward animals commonly observed by people [138]. Grossly, multifocal necrosis affecting many organs is observed, including the liver, kidneys, pancreas, spleen, lymph nodes, and thymus. Fibro-necrotic plaques are present on many organs in both pleural and peritoneal cavities. The main histopathological lesions are represented by systemic vasculitis and perivasculitis. Superficial erosions and ulcers of the gastric mucosa have also been frequently reported. A differentiating feature of the cheetah virus compared to other FCoV strains was the lack of cell fusion (syncytia) in studies performed in vitro [134].

Mountain lions *(Puma concolor)* are susceptible to both FeCV and FIPV. Surveys for CoVs have documented an antibody prevalence of 28% in California mountain lions [138,139]. Small and large intestine are mainly affected together with mesenteric lymph nodes, and vasculitis with fibrinoid necrosis is generally detected at histology. In some cases, the heart can be diffusely involved with vascular necrosis and necrotizing myocardial changes. By means of IHC, FCoVs were detected in the heart, in the intestine, and in the kidney.

In African lions (*Panthera leo*), FIP has been diagnosed and is typically characterized by the involvement of the eye. Eyes show irregular pupil margins, uveal edema, small white collars on the iris, and a gelatinous white membrane covering the retina. At histology, the ocular lesions show bilateral panuveitis with retinal detachment. The predominant infiltrating cells are lymphocytes and plasma cells with scattered macrophages. These cells infiltrate the tissues diffusely or sometimes target small veins. The iris’ pigmented layer is lost, with scattered melanophages in the thickened iris and retina. Eosinophilic proteinaceous exudate accumulates in the anterior chamber. IHC proves FCoV antigen in macrophages. The brain shows dilated perivascular spaces, mainly in the white matter, but no FCoV-positive macrophages are usually detected [140].

In wild cats (*Felis silvestris*), CoV-associated lesions have been described in two main outbreaks registered from 1982 to 1992 in Scotland [141] and were noted in the liver, kidneys, intestine, brain, lungs, heart, and lymphoid tissues. The intestinal wall is often thickened, and necrotic lesions are evident on the surface of many organs. Patchy consolidation of the lungs is also reported. At histology, the serosal surfaces of the liver, spleen, intestine, heart, and lungs are usually markedly thickened by fibrin deposition and a diffuse leucocytic infiltration. Blood vessels, particularly medium-sized arteries, and veins show marked thickening of the tunica adventitia, with infiltration of macrophages, lymphocytes, and neutrophils. In the lungs of one case, there was acute inflammation of alveoli with marked intra-alveolar hemorrhage. Numerous macrophages were present in alveolar and bronchiolar lumen, and marginated mononuclear cells were present in the pulmonary veins and arterioles. Sequential stages of hepatic necrosis were associated with hepatocyte syncytial formation and FIPV was detected by IHC, predominantly in macrophages in the lungs and around vessels in all cases.

### 3.9. Wild Ruminants

Bovine-like CoVs, all currently included within the *Betacoronavirus* (subgroup 1), have been detected in the enteric and/or respiratory tract of wild ruminants, and they are widely distributed in captive and free-range wild ruminant species all over the world.

The majority of bovine-like CoVs have confirmed a close relationship with different BCoV strains that induce gastroenteritis in neonatal calves and lactating cows and respiratory disease complex in growing and steer calves.

It is nowadays generally accepted that bovine-like CoVs are host-range variants of BCoV, which is able to cross the interspecies barriers on a regular basis. Transmission of BCoV variants from cattle to other ruminants and vice versa allows persistence of the infection in nature, recurrent emergence of epidemics, and continuous evolution of the virus. In particular, wild ruminants are not confined to discrete geographic regions and are always moving to seek new pastures, escape from predators, and find mates. Human activities, including—but not restricted to—deforestation, over-hunting, and game farming, also force wild animals to change habitats. The continuous movement of wild animals promotes virus transmission to new terrestrial areas, and, importantly, provides a window for the virus to adapt to new hosts and develop into novel strains.

Bovine-like CoVs have been identified in wild or domesticated ruminants and a brief description of available information follows.

Several species of deer—sambar deer (*Cervus unicolor*) and white-tailed deer (WTD; *Odocoileus virginianus*), sika deer (*Cervus nippon yesoensis*), caribou (*Rangifer tarandus*), and water deer (*Hydropotes inermis*)—show gastroenteritis and harbored CoV, which was antigenically (cross-neutralizing) indistinguishable from BCoV and able to experimentally infect calves [73,89,133,142].

Waterbuck antelopes (*Kobus ellipsiprymnus*) manifest winter dysentery with a Bovine-like CoV detected in fecal samples by TEM and BCoV-specific ELISA, antigenically (cross-neutralizing) indistinguishable from BCoV and able to experimentally infect calves [143].

Giraffes (*Giraffa camelopardalis*) also present Bovine-like CoV-associated gastroenteritis [73,143,144].

Llamas (*Lama lama*) show gastroenteritis and were susceptible to experimental infections with MERS-CoV [145,146,147], whereas Alpaca (*Vicugna pacos*) are infected by a specific *Betacoronavirus* manifesting gastroenteritis and respiratory disease [145] and by a specific *Alphacoronavirus* associated with mild respiratory disease [148].

Dromedary camels (*Camelus dromedarius*) can be naturally or experimentally infected with MERS-related CoVs (recovered most commonly from nasal swabs but also fecal swabs, rectal swabs, and lung tissue). These viruses cause mild upper respiratory tract infection with or without symptoms, including nasal and lacrimal discharge, coughing, sneezing, elevated body temperature, and loss of appetite. In the Middle East, as well as in North and East Africa, there is >90% seroprevalence to the virus [90,149,150,151,152]. The last strain detected in the United Arab Emirates and consequently named dromedary camel coronavirus UAE-HKU-23 (DcCoV UAE- HKU23), is slightly divergent from other BCoV-like viruses, despite still being responsible for similar gastroenteritis [153].

Water buffalo (buffalo calves) can be infected with the bubaline coronaviruses (BuCoVs) that is strictly related to BCoV at the genetic level, but displays some unique biological properties, such as poor growth on MDBK cells and lack of HA activity using chicken erythrocytes. The infection is characterized by a severe gastroenteritis, with enlargement of the mesenteric lymph nodes and gallbladder and congestion/hemorrhages in the small and large intestine that were filled with abundant watery content. By conventional Reverse Transcriptase-Polymerase Chain Reaction (RT-PCR), bovine-like CoV RNA can be detected in the intestinal content of the dead animals as well as in fecal samples from calves with diarrhea [154,155].

Elks or wapiti (*Cervus elephus canadensis*) are able to harbor CoVs antigenically (cross-neutralizing) indistinguishable from BCoV, and able to infect calves in an experimental environment. The reported postmortem findings are enteritis and pneumonia as the leading causes of death in neonatal elks (under three weeks of age) and elk calves (between three weeks and one year of age) [89]. In one case report, interstitial pneumonia and enteritis were diagnosed and allegedly related to CoVs. After the inoculation of elk fecal samples into HRT-18 cells, syncytia formation was observed [156].

Musk oxen (*Ovibus moschatus*) BCoV seropositivity was described in the past in association with watery diarrhea [157]. More recently, Sitatunga (*Tragelaphus spekei*), Wisent (*Bison bonasus),* Hymalayan tahr (*Hemitragus jemlahicus*), and Nyala (*Tragelaphus angasii*) also presented with severe bloody diarrhea (winter dysentery) with weakness, depression, anorexia, and dehydration, and Bovine-like CoVs were detected in fecal samples [143].

### 3.10. Other Wild Animals

Mink epizootic catarrhal gastroenteritis (ECG) was first described in 1975 [158], and later, several millions of minks were reported to be affected in different countries (the USA, Canada, Scandinavia, PR China, and Russia). Usually, infected minks become anorexic and develop mucoid diarrhea within 2–6 days; however, CoV-like particles have occasionally been demonstrated in feces from clinically healthy minks. Due to anorexia, infected minks lose body condition and fur quality, which is of economic concern to the minks’ breeders. CoV infection was confirmed by Transmission Electrone Microscopy (TEM) for ECG. Other enteric viruses such as rotavirus, parvovirus, and calicivirus were suspected to enhance the disease’s severity. Millions of minks were affected in 2011 by a severe acute catarrhal gastroenteritis that was most likely suspected to be a CoV [159].

Recently, SARS-CoV-2 infection and pathological findings were described in minks farmed in the Netherlands in two separate but nearby houses. The symptoms were mostly limited to watery nasal discharge, but some animals showed severe respiratory distress. Thirty-six recently dead animals were collected (18 per farm) and underwent postmortem examination. Twenty-eight animals had diffusely dark to mottled red, wet lung lobes that did not collapse at the opening of the thoracic cavity, suggesting interstitial pneumonia. Histology was performed on seven of these 28 minks, which presented a severe diffuse interstitial pneumonia with hyperemia, alveolar damage, and atelectasis. In the samples collected from all 36 necropsied animals, viral RNA was detected in all tracheal swabs (100%) and in 34 of the 36 (94%) rectal swabs [160].

Infection with an enteric CCoV has only been sporadically detected in wolves (*Canis lupus lupus*), but lesions have not been described [161,162]. Similarly, in red foxes (*Vulpes vulpes*), CCoV infection causes mild, self-limiting enteritis followed by rapid recovery [163]. SARS-CoV RNA was detected in naturally infected red foxes from a live animal market [164].

In raccoons (*Procyon lotor*), fibrinous gastroenteritis affecting the small intestine with diffuse blunting and fusion of intestinal villi and associated with mild bronchopneumonia has been described, in which TEM demonstrated both CoV and parvovirus particles within the feces. SARS-like CoV strains were found to be widespread in raccoon dogs (*Nyctereutes procyonoides)*, which are now suspected to be an intermediate host [165,166,167].

Eurasian otters (*Lutra lutra*) and common genets (*Genetta genetta*) have shown mild, self-limiting enteritis followed by rapid recovery associated with isolation of CoV-like particles [163].

SARS-like CoV strains were found to be widespread in masked palm civets (*Paguma larvata*) suspected to be intermediate hosts [165]. Full-genomic comparative analysis has shown that SARS-like CoVs isolated from palm civets are under strong selective pressure and are genetically most closely related to SARS-CoV strains infecting humans early in the outbreaks [168].

In spotted hyenas *(Crocuta crocuta),* FCoV type II and CCV were identified from stools, but no lesions were described [169,170]. Similarly, CoV was genetically identified in the feces of silver-backed jackal (*Canis mesomelas*) with no described lesions [170].

In European hedgehogs (*Erinaceus europaeus),* hedgehog coronavirus 1 (subgenus Merbecovirus), referred to as Erinaceus CoV (EriCoV), was not associated to disease, so that the Western European hedgehog is considered a potential reservoir host [171,172,173]. Similarly, in amur hedgehogs (*Erinaceus amurensis*), hedgehog coronavirus HKU31 (Ea-HedCoV HKU31) was detected with no description of associated lesions [174]. Additionally, in Asian house shrew (*Suncus murinus*), a novel member of the genus *Alphacoronavirus*, named Wénchéng shrew virus (WESV), was found, although not associated with any form of disease. As such, shrews may play an important role in the evolution and transmission of coronaviruses, among animals, or as a source of zoonotic infections [174].

### 3.11. CoVs in Non-Human Primates

Human CoV (HCoV)-OC43, *Betacoronavirus* (clade 1), which is an endemic human pathogen causing episodes of the common cold in humans worldwide, has been registered as responsible for sporadic coughing and sneezing, mainly in morning hours in a group of wild chimpanzees (*Pan troglodytes verus*) living in the Taï National Park, Côte d Ivoire [175]. PCR and sequencing identified the HCoV-OC43 in 14 out of 59 samples collected from 11 individuals, including those where symptoms were consistently reported. The detection in feces exclusively during the outbreak supports the hypothesis of this coronavirus being responsible for the observed mild respiratory symptoms. HCoV has been reported as responsible for respiratory anthroponosis in wild great apes.

No data is supporting host-specific CoVs in great apes and other primates, but this report hints that anthroponotic transmission of HCoV-OC43 can result in respiratory disease in chimpanzees, revealing yet a new interface in coronavirus host switching.

### 3.12. CoVs in Marine Mammals

Beluga whale CoV SW1 (BWCoV-SW1) was associated with one subject’s death after a short medical illness characterized by generalized pulmonary disease and terminal acute liver failure [176]. The liver demonstrated a diffusely increased friability with areas of necrosis. Conventional TEM was performed on liver tissue, and numerous round viral particles measuring 60 to 80 nm with a core of approximately 45 to 50 nm were identified in the cytoplasm of hepatocytes, but this was insufficient to identify the virus. Pan-viral microarray assay (ViroChip) was used to directly recognize this novel CoV from primary animal tissues (liver).

In fecal samples of 3 Indo-pacific bottlenose dolphins, the BdCoV HKU22 strain was identified. Comparative genome analysis showed that BdCoV-HKU22 and BWCoV-SW1 have similar genome characteristics and structures, displaying a 98% nucleotide sequence identity to each other [177].

### 3.13. CoVs in Laboratory Animals

Mice, rats, guinea pigs, and rabbits represent the most common laboratory animal species used in biomedical research facilities [178]. CoVs affecting rodents, initially referred to as Murine CoV, belong to the genus *Betacoronavirus*, subgenus *Embecovirus*, where rodent, human, and bovine CoVs are included. Rodents are thought to play a significant role in the evolution of CoVs, and it has been suggested that rodent CoVs are the ancestors of embecoviruses—a subgenus within *Betacoronavirus*—affecting other animal species [28], including humans (CoV-HKU1). Murine CoVs include the mouse hepatitis virus (MHV) and rat coronaviruses (RCoV), namely sialodacryoadenitis virus (SDAV) and Parker’s RCoV (PRC). Recently, new species of *Alpha* and *Betacoronaviruses* (LRNV, LAMV, LRLV, and HKU24) have been identified in rodents in Europe and China, but they had not been associated with lesions [179,180,181,182].

MHV is one of the most studied CoV in animals and is also used as a model system to study multiple sclerosis [183] and autoimmune hepatitis [184] in humans. First identified in 1949, MHV is one of the most common viruses among wild and laboratory mice worldwide [185,186]. It comprises several different strains that cause different diseases. The severity of the lesions depends on virus strain as well as on host factors, such as age, immune status, and genotype [187,188]. Typically, MHV disease is not clinically evident, and most infected animals are asymptomatic [187]. This represents a major problem in animal facilities, where subclinical MHV infection could alter experiment reliability [189]. Strains of MHV are classified into two major groups: polytropic and enterotropic biotypes. Polytropic strains initially replicate in the nasal respiratory epithelium and disseminate to other organs via the blood, lymphatic system, and olfactory nerve [186,187], whereas enterotropic strains target intestinal mucosal epithelium and are characterized by minimal or no dissemination to other tissues [187].

Polytropic MHV strains may be associated with necrotic foci on the liver surface, which can become hemorrhagic in immunodeficient mice [21], fibrin deposition, and mild infiltration of mononuclear inflammatory cells [188]. Mice can also develop hepatic nodular hyperplasia with fibrosis and parenchymal collapse. Microscopically, affected organs such as liver, spleen, lymph nodes, thymus, bone marrow, and gut-associated lymphoid tissue present multifocal to coalescing areas of acute necrosis as well as syncytia of parenchymal cells and endothelial cells. Microthrombi in liver sinusoids might be present [19]. However, syncytia may not be so evident in immunocompetent mice [187]. MHV strain MHV-S can cause mild olfactory mucosal necrosis, neuronal necrosis of olfactory bulbs, and interstitial pneumonia [190,191]. Mice infected with MHV-1 strain showed interstitial pneumonia, hyaline membrane and fibrin deposition, infiltration with lymphocytes, macrophages, and neutrophils, and death [190]. MHV-1 infection of A/J mice provides a mouse model for the pathogenesis of SARS-CoV in humans [21].

Strains MHV-JHM and MHV-A59 are predominantly neurotropic, and lesions are often seen in neonatal and immunocompromised mice. They may show vestibular signs and posterior paresis. Lesions are mainly seen in experimental infections and include necrotizing nasoencephalitis, meningoencephalitis, optic neuritis, loss of retinal ganglion cells, retinal vasculitis and perivasculitis, neuronal cell loss and degeneration, apoptosis with infiltration of macrophages, neutrophils, and natural killer cells, as well as syncytia [21,190,192,193,194,195,196,197,198,199]. In survivors, chronic demyelination is often seen, and for this reason, infected mice are used as an animal model for human multiple sclerosis [200]. MHV 3 is neurotropic, depending on the strain of the mice infected. Lesions are characterized by meningitis, ependymitis, and leukoencephalitis, followed by hydrocephalus and, between 60 and 130 days post-infection, chronic thrombotic vasculitis of meningeal and parenchymal vessels at the brainstem level [201,202].

In a study, gamma interferon (IFN-γ)-deficient mice developed pleuritis, peritonitis, pneumonia, and meningitis, suggesting a role of IFN-γ in the pathogenesis of MHV [203].

Lesions due to enterotropic MHV strains, in neonatal mice, are primarily located in the terminal small intestine, cecum, and colon. They are characterized by severe necrotizing enterocolitis with villous attenuation, syncytia of enterocytes and endothelial cells of mesenteric vessels, leukocytic infiltration, and occasionally hemorrhages [187,204]. Eosinophilic intracytoplasmic inclusions may be present within enterocytes [187]. Lesions in adults are minimal with enterocytic syncytia predominantly located in the cecum and ascending colon. Adult nude mice may develop chronic hyperplastic typhlocolitis and mesenteric lymphadenopathy [187]. Rarely, some enterotropic MHV strains can disseminate and cause hepatitis and encephalitis [187]. Mice that have recovered may develop residual lesions, such as reactive hyperplasia of spleen and lymph nodes, hematopoietic hyperplasia, perivascular cuffing in the lungs and brain, foci of inflammation in the liver, and intestinal mucosal hyperplasia [187].

PRC and SDAV are the two natural CoVs infections affecting rats, with SDAV being one of the most common infections in laboratory rats worldwide [205,206,207,208,209]. They are antigenically related to MHV, BCoV, and HCoV strain OC43 [209,210]. Although they are considered two different viruses, they produce overlapping lesions. Typically, PRC is associated with respiratory lesions, whereas SDAV causes primarily an inflammation of salivary and lacrimal glands but can also cause pulmonary disease in young rats [187,211,212]. SDAV is characterized by high morbidity and negligible mortality [187].

Lesions of respiratory disease associated with PRC include necrotizing rhinitis, laryngitis, tracheitis, bronchitis, all with hyperplasia, flattening, and sloughing of respiratory epithelial cells. Lymphocytes, plasma cells, macrophages, and neutrophils are present [187,213,214,215]. Interstitial pneumonia with leukocyte infiltration in the alveolar wall and type I pneumocytes hyperplasia can arise [212,213]. Also, type II pneumocytes can be a target of SDAV in respiratory infections [212]. Athymic nude rats may develop suppurative rhinitis and bronchopneumonia [187]. Rat coronavirus infection can be used as a spontaneous animal model to study HCoVs that cause non-fatal respiratory diseases [212]. The classical clinical signs associated with SDAV are depicted as cervical swelling due to edema and inflammation of parotid and/or submandibular salivary glands, blepharospasm, sniffling, epiphora, and often chromodacryorrhea (i.e., dark red crusts around the eyes) [187,216]. Reduced fertility may be present [206]. In general, all the salivary and lacrimal glands are enlarged by edema and inflammation. Microscopic lesions of salivary and lacrimal glands during the acute phase of infection are characterized by necrosis of the ductal epithelium, interstitial edema, infiltration with lymphocytes, plasma cells, macrophages, and neutrophils [216,217,218]. Starting from 7 days post-infection, during the recovery phase, the salivary and lacrimal glands present a nonkeratinizing squamous metaplasia of ductal and acinar epithelium, mild infiltration with mononuclear inflammatory cells, as well as periglandular, interlobular, and ductal fibrosis [216]. Lesions to eyes, such as keratitis sicca, hyphema, impaired intraocular damage, and megaglobus have been reported [211]. Additionally, it has been reported that SDAV can experimentally cause lesions to the CNS in animals during their first week of age. Microscopic lesions are represented by acute necrotizing encephalitis associated with minimal inflammatory response, that in survivors later develop as loss of brain substance, astrogliosis, gitter cells’ infiltration (i.e., activated microglia), and occasional focal areas of mineralization. In close proximity of malacic areas, endothelial cells lining vessels are often hypertrophic and hyperplastic. Lesions are mainly located in the cortical regions of the frontal, parietal, occipital, and temporal lobes, diencephalon, and hippocampus. Rarely, lesions extend to the olfactory bulbs, mesencephalon, and cervical region of the spinal cord [206].

Recently, a novel rat coronavirus has been discovered in China and has been named as HKU24. However, to date, although an enteric tropism has been suggested, there is no evidence that this virus causes disease in rats [181].

A coronavirus-like syndrome has been reported in guinea pigs that presented anorexia, wasting, and diarrhea. Lesions were represented by the accumulation of mucoid material within the small intestine. Microscopically, animals were characterized by acute to subacute necrotizing enteritis with blunting and fusion of villi, necrosis, and enterocytes’ syncytia. Lesions were mainly located in the distal ileum [219].

Guinea pigs, experimentally infected with SARS-CoV, developed lesions in the lungs, characterized by thickness of alveolar walls due to type II pneumocyte hyperplasia and to the accumulation of fibrin within the interstitium, hyaline membrane deposition, and scattered fibroblast proliferation [220].

In rabbits, the only coronavirus responsible for natural infection is the Rabbit Enteric Coronavirus (RECoV), a *Betacoronavirus* that has been reported in young rabbits from commercial rabbitries and barrier-maintained laboratories in Europe, Canada, and China, and it is probably present in the USA as well [221,222]. RECoV usually causes transient mild diarrhea in young rabbits. Gross lesions are generally mild, characterized by cecum distension containing watery, white to tan feces [221].

RECoV microscopic lesions, like other CoVs, are only present in the small and large intestine and are consistent with villous blunting, vacuolation and necrosis of enterocytes, mucosal edema, and polymorphonuclear and mononuclear cell infiltration. Enterocytes lining villi at the level of the gastro-intestinal associated lymphoid tissue (GALT) may contain cytoplasmic vacuoles, crypts can be hypertrophic, and epithelial cells covering lymphoid follicles may undergo necrosis. Infected animals show increased mitotic figures (regeneration) and cellularity in intestinal crypts [221,222].

### 3.14. CoVs in Birds

CoVs affecting avian species (AvCoV) are classified into two genera: *Gammacoronaviruses*, within which the main representatives are AvCoVs [223], and *Deltacoronaviruses*, identified for the first time in 2009 in passeriform birds and affecting avian species and pigs [224,225].

An exception to this seems to be a Bovine CoV (*Betacoronavirus*), which was recently demonstrated to induce symptoms (diarrhea and enteritis) if inoculated in turkeys [226,227].

AvCoVs affect both domestic and wild birds and include infectious bronchitis virus (IBV) of chickens and other antigenically similar viruses infecting other domestic birds such as turkeys, guinea fowls, pheasants, peafowls, quails, partridges, blue-winged teals, pigeons, mallard ducks, and gray-lag gooses [223,228,229]. Studies from the last two decades trying to understand their role in domestic bird infections have also shown the presence of AvCoV strains in healthy wild birds [230,231]. The variation in host range, tissue tropism, and pathogenesis of avian CoVs is mainly due to variations in the S glycoprotein and lead to different antigenic forms, serotypes, or variant strains [232,233]. New antigenic virus variants continue to appear, leading to an inconsistent nomenclature and classification of virus strains [233].

### 3.15. Domestic Birds

Infectious bronchitis (IB) is the main disease caused by AvCoVs in poultry [234] and IBV belongs to the *Gammacoronaviruses*, subgenus *Igacovirus* [235]. The virus, with different strains, can infect domestic fowl (*Gallus gallus*), but also other gallinaceous and not-gallinaceous domestic species [236,237,238]. Additionally, some CoVs detected from turkeys, peafowls, and other birds were highly homologous to IBV [239,240,241]. In many studies, IBVs have been detected in healthy wild birds, demonstrating their role as vectors between domestic and free-living birds [224]. The disease was firstly reported in 1931 in the USA as a respiratory disease. Nowadays, IBV is ubiquitous in intensive poultry production in most parts of the world in both industrial and backyard chickens [224,242]. IBV causes huge economic losses: high mortality, poor body weight gain in broilers, and decreased egg production in layers [242,243]. In contrast to virus species belonging to the *Alpha*- and *Beta-coronaviruses*, which occur as only one or two different serotypes, there are many different serotypes of the chicken *Gammacoronavirus* IBV [244,245]. The control of IBV in the field is hampered by the many different strains circulating worldwide and the limited protection across strains due to serotype diversity [232]. IBV causes a highly contagious disease affecting the respiratory tract and, depending on the strain, other tissues, including the reproductive and urogenital tract [232].

The host factors involved in IBV attachment are alpha2,3-linked sialic acid (the main one), specific lectins, heparan sulfate, and cellular furin [232]. The role of macrophages in IB has been investigated in the last decade. It has been demonstrated that this type of inflammatory cells can be infected by IBV, both in vivo and in vitro, resulting in productive replication and apoptotic processes, through both intrinsic and extrinsic pathways [246]. Macrophages affected by IBV produce IL-1β, suggesting the potential roles of these mediators in the host responses to IBV infection [247]. Reddy and collaborators demonstrated that a nephro-pathogenic strain of IBV could replicate in peripheral blood monocytes leading to viremia [248]. The formation of multinucleated syncytia was demonstrated in cell cultures, and their formation depended on the strain of IBV [249,250].

Respiratory airways show inflammation with catarrhal exudate. Also, lung congestion, catarrhal pneumonia, and fibrino-suppurative pneumonia, particularly in cases complicated by *E. coli*, are observed. Additional lesions are represented by catarrhal or fibrinous aerosacculitis, catarrhal conjunctivitis, interstitial nephritis (by nephrotropic strains) with urates precipitation, atrophy of the oviductum, and peritonitis (in false layer syndrome) [242,251,252].

The respiratory airways are characterized by inflammation of the mucosa with heterophilic and lymphocytic infiltration, loss of cilia and sloughing of epithelial cells, epithelial cell hyperplasia, and hypertrophy of the mucosal gland. In the lungs, pneumonia with infiltration of lymphocytes and heterophils, fibrinous exudate, congestion, hemorrhages, as well as edema, epithelial desquamation, and fibrinous exudate in the air sacs are evident. Kidneys show interstitial nephritis with lymphocytic and heterophilic infiltrate, hemorrhages, tubular degeneration and necrosis, edema of Bowman’s capsule, and dilated renal tubules with urate crystals and cast. The liver is often characterized by congestion of the central vein and hepatic sinusoids. Also, portal veins show thickening and hyalinization of the wall beside focal aggregation of lymphocytes and heterophils, degeneration, and necrosis of hepatic cells, with distortion of hepatic cords and infiltration of inflammatory cells. Thickening and hyalinization of central arterioles with endotheliosis and depletion of lymphocytes are seen in the spleen. The brain is characterized by perivascular lymphocytic cuffing, degenerated neurons, satellitosis, and neuronophagia [242,250,253]. IBV can infect glandular epithelial cells of the proventriculus [254] as well as histiocytes, lymphoid cells, and enterocytes lining the cecal tonsils [255]. However, this does not result in significant clinical gastrointestinal disease. IBV can also infect the Harderian gland [253,256,257].

Coronaviruses in pheasant (*Phasianus colchicus*) (PhCoVs) are associated with respiratory disease and more often with nephritis [223,258,259]. PhCoVs belong to the genus *Gammacoronavirus* and subgenus *Igacovirus* [233,235] and have a high degree of genetic similarity with chicken and turkey CoVs [258,259,260,261]. Han and collaborators reported different evolution patterns and tissue tropisms between different PhCoVs [262]. Cavanagh and collaborators confirmed that CoVs that naturally infect pheasants are genetically similar to IBV, but they do not cause disease when inoculated experimentally in chickens, even if antibodies are produced [243,258,260]. Additional studies of the complete genomic sequence are necessary to better understand the relation between the pathogenicity of IBVs in pheasants and clarify the link between PhCoVs and IBVs [262]. Outbreaks associated with CoVs infection in pheasants were reported for the first time in the 1980s. PhCoV was isolated in the UK [260,261,263] and in China [262]. Grossly, the celomic cavity usually shows visceral gout, swollen and pale kidneys, with distended ureters with urates and urolithiasis [260,263]. Welchman and collaborators reported a significant association between sinusitis and CoVs detected by PCR, but the association was greater between sinusitis and Avian Pneumovirus (APV) serology [264]. At histology, a moderately severe interstitial nephritis has been described [259,263].

Pigeon (*Columba livia*) CoVs (PiCoVs) belong to genus *Gammacoronavirus* and subgenus *Igacovirus* [233,235] and have been isolated in Australia [238], Norway [230], and China [265]. PiCoV infections were reported in association with clinical signs of ruffled feathers, dyspnea, presence of mucus in the beak [238], and pancreatitis [236,265]. Depression, weakness, wheezing, watery eyes, and tracheal rales were also described, with the major gross lesion represented by severe pancreatitis. Pancreatic tissue samples from sick pigeons were extracted, homogenized, and inoculated in 10-day-old specific pathogen-free embryonated eggs. The allantoid fluid containing the virus was inoculated in 30-day-old pigeons and 15-day-old chickens, which developed lesions mainly in the pancreas and the lungs. The virus was closely related to IBV strains and genetically distinct from Turkey CoVs. The role of the virus in upper respiratory tract infections has not been thoroughly examined [236].

Turkey (*Meleagris gallopavo*) CoV (TCoV) is a worldwide diffuse virus that causes diarrhea and weight loss with postmortem lesions consistent with severe enteritis with flaccid pale and thin-walled intestine with watery content [227]. Histological examination of different zones of the intestinal tract of the TCoV-infected turkeys showed that the virus targets the lower part of the intestinal tract with an abundance of viral antigen being present in epithelial cells of the ileum, caecum, and bursa of Fabricius [266]. Viral antigen was also detected in dendritic cells, monocytes, and macrophages in the affected areas, indicating a potential for TCoV to replicate in antigen-presenting cells [266]. In the small intestine of affected turkeys, a different study identified multifocal marked pseudostratification of the epithelium, villous atrophy, infiltration of the epithelium by small numbers of mainly lymphocytes, the formation of lymphoid aggregates in the lamina propria and submucosa, and multifocally scattered infiltration of moderate numbers of plasma cells and heterophils [232,267]. This tropism for the enteric tract is consistent with a change in the S protein’s ability to attach, which is mediated by non-sialylated type 2 poly-LacNAc structures on N-glycan cores. This is markedly in contrast with the sialic acid-dependent binding of other AvCoVs inducing primarily respiratory tract disease, including IBV [232].

It was recently demonstrated that BCoVs can infect turkeys, causing severe enteritis, which again highlights the capacity of CoVs to spillover [226]. In 2008, the genome of a CoV causing severe enteritis in turkeys was sequenced [268] and its origin was ascribed probably to a mutation of the IBV of chickens [269] that allowed the virus to acquire tropism for the intestinal tract. Additionally, a recombinant strain of IBV with TCoV has been recently identified [270].

A specific coronavirus was firstly isolated in the guinea fowl (*Numida meleagris*) in France, causing severe enteritis and occasionally pancreatitis. Although the disease has been noted since 1980, virus isolation and characterization were obtained only in 2014 [229]. The same virus was also isolated in China and Brazil [223]. According to the authors, the most likely evolution of this virus is consistent with a recombination of IBV with another AvCoV, allowing adaptation to a different avian species [229] and acquiring tropism for the enteric tract due to the attachment capacity of the viral S1 proteins [271].

Other birds have been found infected by specific non-recombinant CoVs, including the Japanese quail (*Coturnix japonica*), where CoVs caused symptoms in kidneys, gastrointestinal (enteritis), and reproductive tract [272,273] in Italy and Brazil. Additionally, specific CoVs seem to cause dilation of the proventriculus [274] in the green-cheeked amazon parrot (*Amazona viridigenalis*) and have been isolated in Italy and China from grey-lag geese (*Anser anser*) and mallard ducks (*Anas platyrhynchos*) [223].

### 3.16. Wild Birds

After the SARS event in 2002/2003, the attention on CoVs in wildlife, including wild birds, has increased significantly [228]. Wild bird species serve as a natural reservoir of many emerging zoonotic pathogens. Among factors that make birds an excellent reservoir of numerous different pathogens and a bioreactor contributing to their variability, there are the high biodiversity of bird species and their ecological traits such as gathering/grouping during feeding and roosting, and most importantly, their capability to fly long distances [224]. Surveillance of wild birds is pivotal in order to assess their role in domestic birds’ infections and the relative prevalence of IBV strain variants for a better risk assessment and risk management of these viruses [230,231,275]. It has been confirmed that wild birds can carry gamma-coronaviruses asymptomatically and could be a genetic reservoir for future emerging pathogenic CoVs [228].

### 3.17. CoVs in Fish

In aquatic species, there is a lack of information concerning the classification of various novel viruses isolated in the last years.

The only mentionable CoV is the White Bream Virus. This virus can infect the white bream (*Blicca bjoerkna*) [276] without any symptoms, and its genetic sequencing has revealed a new subfamily of Coronaviruses called *Piscanivirinae.* Specifically, this virus belongs to the genus *Bafinivirus,* which seems to be a primitive form of the mammal CoVs [277]. This virus can also infect other cyprinids like goldfish (*Carassius auratus*), tench (*Tinca tinca*), and grass carp (*Ctenopharyngodon idella*), causing hemorrhages on the gills and skin, or ulceration of the skin. It was also isolated from a dead crucian carp (*Carassius carassius*) that presented nonspecific symptoms like exhaustion, reduced food intake, increased mucus production, pseudofeces, and petechiae [277].

### 3.18. CoVs in Reptiles and Amphibians

CoVs in reptiles were observed only in the feces of four Nile crocodiles, using negative staining TEM [278]. No description of the pathological lesions is available in the literature.

There is no literature about Coronavirus in amphibians.

### 3.19. CoVs in Humans

Far from the aim of this review is to cover all information regarding CoVs in humans. Briefly, the three highly pathogenic coronaviruses (SARS-CoV-2, SARS-CoV, and MERS-CoV) cause severe respiratory syndrome in humans, often when comorbidities are present. The other four coronaviruses (HCoV-NL63, HCoV-229E, HCoV-OC43, and HKU1) only cause mild upper respiratory disease or remain harmless. Human CoVs belong to *Alphacoronavirus* (HCoV-NL63 and HCoV-229E) and *Betacoronavirus* (SARS-CoV-2, SARS-CoV, MERS-CoV, HCoV-OC43, and HKU1) and genomic data from the literature allow to consider all human CoVs to have animal origin [12]. Gastrointestinal clinical symptoms and dermatitis have been less frequently described in non-fatal cases, while encephalitis with nervous symptoms has been reported sporadically in fatalities [279,280,281].

SARS-CoV pathology and pathogenesis have been broadly studied after its spread in November 2002. The pathogenesis is multifactorial and highly complex, involving direct cytopathic damage of cells, immune system derangement, downregulation of lung-protective angiotensin-converting enzyme 2, and genetic factors [282]. Several cells/organs can be affected (e.g., respiratory, intestinal and renal epithelium, neurons, immune cells), the main target being alveolar pneumocytes in which syncytial cells’ formation can be seen [282,283]. Thrombosis and vascular damage have been reported accompanying fibrinous pulmonary exudation in acute severe cases followed by chronic fibrotic damage [283]. Comparable lesions, although less severe, have been documented in the few studies regarding MERS [284,285,286,287].

Documentation of SARS-CoV-2 lesions in fatal cases is scarce due to the low number of postmortem examinations performed, making the pathogenesis of the disease still under investigation [279]. In studies performed on selected small case series, the main finding is represented by diffuse alveolar damage occasionally accompanied by microthrombi and vascular damage, the latter being described systemically in some cases [279,280,281,288,289,290,291,292]. Rarely, multinucleated pneumocytes have been detected [288]. Immune depletion is also frequently observed, and myocarditis/endocarditis, nephritis, and encephalitis have also been reported. CoV particles and antigen have been detected by TEM and IHC in the respiratory system (alveolocytes and tracheal epithelium), kidneys, and gastrointestinal tract both within epithelial cells and macrophages.

## 4. Discussion

The tremendous amount of animal diseases caused worldwide by a variety of CoV strains in several species indicate the vast spreading of CoVs in the ecosystem and their ability to change, adapt, and progressively cause new animal diseases over time. The adaptation during cross-species jumps in different species including domestic and wild mammals, as well as birds, may play a role in enabling viral spillover from natural hosts to humans. Moreover, infection of domestic species from human CoVs has also been partially documented and recently demonstrated for SARS-CoV-2 [293], indicating a potential mutual role in the transmission of the infection.

Extensive genomic studies of both human and animal CoVs allow for a better understanding of the origin and evolution of pathogenic CoVs, pivotal for disease control and treatment, and hopefully helping in avoiding the new wide spreading of life-threatening diseases. In the One-Health age, an increased animal-to-human transmission is already evident of viral pathogens such as Ebola, influenza viruses, Hendra, Nipah, and CoVs. Ecosystem changes, including climate changes, urbanization with increased human population, and cultural and social changes, as well as secular traditions [294], account for this new spreading of zoonotic epidemics of which we should regrettably expect more in the future.

For these reasons, animals, humans, and the environment should be considered as part of the same scenario and a better understanding of the interaction between the different components could help in preventing and controlling any future spill-over towards the human sphere. A proper management of environmental factors by increasing attention to land usages aimed to preserve biodiversity, to prevent a wild/domestic interaction, and to avoid stressful conditions to wild species reservoirs seems to be a good approach in reducing spill-over risks. This stresses the urgent need of multidisciplinary approaches and a constant monitoring of the wild animal sphere. A proper surveillance program including constant reporting and investigations on dead wild and domestic species could help to anticipate the spread of a similar epidemic.

As previously discussed, CoVs, like other positive-strand RNA viruses, have the ability to manifest and acquire genetic diversity due to some typical features, such as the infidelity of the RNA-dependent RNA polymerase, the high frequency of homologous and heterologous RNA recombination, and the large genomes [295]. This genetic variability confers to CoVs the high potential of evolution that occasionally allows them to overcome species barriers and host specificity [296,297,298].

Some studies indicate that possibly all CoVs are genetically derived from common ancestors residing in bats, which are usually naturally infected and asymptomatic long-lasting reservoir (*Alpha* and *Betacoronavirus*), and in birds (*Delta* and *Gammacoronavirus*) [12,32,295,299]. The different behavior of coronaviruses in bats and birds could also be related to the unique properties of these two groups of animals. The diversity of bats and birds themselves is huge, their flying capacity has allowed them to spread worldwide, and their habits provide frequent opportunities of aggregation [299].

The genomic diversity of CoVs accounts for their variation in species adaptation related to receptor binding ability and, consequently, tissue tropism, producing localized versus systemic diseases affecting different organ systems [12]. As an example, SARS-CoV uses ACE2 as a receptor and primarily infects ciliated bronchial epithelial cells and type II pneumocytes, whereas MERS-CoV uses DPP4 and infects non-ciliated bronchial epithelial cells and type II pneumocytes [12].

As described in the text and summarized in Table S1, in CoV infections, three major organ systems appear to be involved: the respiratory, the alimentary, and the nervous system (Scheme 3, Scheme 4 and Scheme 5). Usually, human CoVs cause mainly respiratory diseases (*Alpha-* and *Beta-coronavirus*), whereas other mammals manifest predominantly gastroenteritis and a less frequent, but typical for some diseases/species, nervous involvement (*Alpha-* and *Beta-coronavirus*). All *Alpha-* and *Beta-coronaviruses* seem to have originated in bats, whereas a separate origin is postulated for avian *Delta-* and *Gamma-coronavirus*, rarely affecting mammals (i.e., pigs) and causing mainly respiratory pathologies [12,32,299].

Interestingly, genomic diversity can not only modify CoVs among species but also within the same species, conferring new ability to spread within the organism. A typical example is the case of FIP, for which risk factors for host and environmental spreading, maintenance, and genetic change associated with increased disease severity are ascribed to animal-to-animal contact and poor infection controls. Additionally, the importance of the genetic background of the host is also relevant, as demonstrated by the similar disease evolution in domestic and wild felids.

Notably, histopathological evaluation of CoVs in animals has underlined similarities with human CoVs, such as the typical alveolar damage and the vascular thrombosis with fibrinous exudation, occasional syncytia formation, depletion of lymphoid organs, and the direct intestinal epithelial damage. As for many other pathological processes (e.g., tumors), animals could therefore not only benefit from human medicine but also represent a model as well as an important ring in epidemiological chains that need to be studied and monitored.

Investigating the lesions and distribution of CoVs can therefore be crucial to understand and monitor the evolution of these viruses as well as of other pathogens in light of the One-Health approach. Unfortunately, mainly in animals, accurate postmortem examinations and histopathological investigations are infrequently performed. However, histopathological characterization, especially in cases with fatal COVID-19, is considered critical to further understand the pathogenesis and transmission of this disease in order to help public health-preventive measures and therapies.

## 5. Conclusions

In conclusion, we believe that further work is absolutely needed in order to better characterize the transmission barrier(s) between CoVs and different animal species, along with the virus- and the host-related factors underlying cross-species jumping within different environments as well as at the level of the various ecological interfaces. Strengthening public health surveillance systems, including veterinary services and wildlife monitoring, could provide early warnings and predict possible future emergencies [300].

## Supplementary Materials

The following are available online at https://www.mdpi.com/2076-2615/10/12/2377/s1, Table S1: Coronavirus-associated diseases in animals and list of lesions in the main affected tissues.
