# Supplementary material for: Pathology of Coronavirus Infections: A Review of Lesions in Animals in the One-Health Perspective
Source: Animals (Basel). 2020 Dec 11;10(12):2377. doi: 10.3390/ani10122377 (PMC7764021; doi:10.3390/ani10122377)
Supplement: Supplementary file 1 [file animals-10-02377-s001.pdf]

animals

MDPI

Supplementary Materials: Pathology of Coronavirus Infections: A Review of Lesions in Animals in the One-Health Perspective

Valentina Zappulli [1], Silvia Ferro [1,*], Federico Bonsembiante [1,2], Ginevra Brocca [1], Alessandro Calore [1], Laura Cavicchioli [1], Cinzia Centelleghe [1], Giorgia Corazzola [1], Steffen De Vreese [1,3], Maria Elena Gelain [1], Sandro Mazzariol [1], Valentina Moccia [1], Nicolò Rensi [1], Alessandro Sammarco [1,4], Filippo Torrigiani [1], Ranieri Verin [1] and Massimo Castagnaro [1]

[1] Department of Comparative Biomedicine and Food Science, University of Padua, Legnaro, 35020 Padua, Italy; valentina.zappulli@unipd.it (V.Z.); federico.bonsembiante@unipd.it (F.B.); ginevra.brocca@phd.unipd.it (G.B.); alessandro.calore.1@studenti.unipd.it (A.C.); laura.cavicchioli@unipd.it (L.C.); cinzia.centelleghe@unipd.it (C.C.); giorgia.corazzola@gmail.com (G.C.); steffen.devreese@studenti.unipd.it (S.D.V.); mariaelena.gelain@unipd.it (M.E.G.); sandro.mazzariol@unipd.it (S.M.); valentina.moccia@phd.unipd.it (V.M.); nicolo.rensi@phd.unipd.it (N.R.); alessandro.sammarco@unipd.it (A.S.); filippo.torrigiani@unipd.it (F.T.); ranieri.verin@unipd.it (R.V.); massimo.castagnaro@unipd.it (M.C.)

[2] Department of Animal Medicine, Productions and Health, University of Padua, Legnaro, 35020 Padua, Italy

[3] Laboratory of Applied Bioacoustics, Technical University of Catalunya, BarcelonaTech, Vilanova i la Geltrù, 08800 Barcelona, Spain

[4] Department of Neurology and Radiology, Massachusetts General Hospital, Harvard Medical School, Boston, 02129 MA, USA

* Correspondence: silvia.ferro@unipd.it

Received: 05 November 2020; Accepted: 09 December 2020; Published: 11 December 2020

Table S1. Coronavirus-associated diseases in animals and list of lesions in the main affected tissues. In black: Main lesions; in grey: Less frequently reported lesions. NR, not-reported; NHP, non-human primate.

	Host species	Name of disease/condition	Viral genus, subgenus	Virus strains/name	Main extension of disease	Main involved tissues and associated lesions				
						Upper respiratory tract	Lower respiratory tract	Alimentary system	Nervous system	Hematopoietic and lymphatic systems/ Urinary system
Pets	Dog		**Alphacoronavirus**	**Canine Enteric CoV type I and II**	Localized			Mild enteritis with villous atrophy. Attenuated low cuboidal to squamous epithelial cells		Lymphoid depletion of Peyer's patches
			Alphacoronavirus	Canine Pantropic CoV type II-a	Systemic		Fibrino-hemorrhagic and necrotizing bronchopneumonia	Hemorrhagic lymphoplasmacytic enteritis with fibrosis,		Lymphoid depletion in spleen, thymus and lymph nodes and gut

**Host species**	**Name of disease/conditio**	**Viral genus, subgenus**	**Virus strains/name**	**Main extension**	**Main involved tissues and associated lesions**				
					**Upper**	**Lower respiratory**	**Alimentary system**	**Nervous system**	**Hematopoietic and**
						associated with perivascular and interstitial edema, vascular necrosis and emphysema	villus blunting, crypt ectasia or necrosis and submucosal and serosal edema Ileocecal intussusception		associated lymphoid tissue
		Betacoronavirus	Canine Respiratory CoV (CRCoV)	Localized	Mild rhinitis with loss of mucosal cilia	Mild tracheobronchitis and presence of peribronchiolar and perivascular lymphoid aggregates in lungs			
**Cat**		Alphacoronavirus	Feline enteric CoV type I and II (FECV)	Localized			Catarrhal and/or hemorrhagic enteritis with villous fusion and atrophy and sloughing of mucosal epithelium		Lymphoid depletion of Peyer's patches
	Feline Infectious Peritonitis (FIP)	Alphacoronavirus	Feline systemic CoV type I and II (FIPV)	Systemic		Pleuritis and pyogranulomatous interstitial pneumonia. Pleural effusions. Granulomatous pleuritis	Fibrinous and granulomatous peritonitis with serous protein-rich abdominal effusions. Pyogranulomatous enteritis and vasculitis	Ependymitis, periventriculitis meningoencephalitis with periarteritis, phlebitis, edema and periventricular reactive astrocytosis, and fibrin perivascular cuffs. Spongiosis and axonal spheroids in the white matter	Lymphoid depletion or focal pyogranulomatous lymphadenitis
**Rabbit**		Betacoronavirus	Rabbit Enteric CoV (RECoV)	Localized			Mild necrotizing enteritis with villous blunting, crypts hypertrophy, vacuolation of enterocytes and mucosal edema		
**Ferret**	Epizootic catarrhal enteritis	Alphacoronavirus, Minacovirus	Ferret enteric coronavirus (FRECV)	Localized			Necrotizing lymphocytic enteritis, with villus atrophy, fusion, and blunting		

		n			of disease	respiratory tract	tract			lymphatic systems/ Urinary system
**Pets**	**Ferret**	Ferret systemic coronavirosis	Alphacoronavirus, Minacovirus	Ferret systemic coronavirus (FRSCV)	Systemic		Pyogranulomatous pneumonia	Pyogranulomatous enteritis (small intestine)	Severe pyogranulomatous leptomeningitis, chorioiditis, ependymitis, and encephalomyelitis, centered on vessels	Multifocal pyogranulomatous lymphadenitis
	**Bovine**		Betacoronavirus	Bovine coronavirus (BCoV)	Systemic	Degeneration and necrosis of epithelial cells in nasal turbinates and trachea. Segmental hyperplasia of the tracheal epithelium, with replacement by cuboidal to flattened epithelium	Thickening of the pulmonary interstitium, necrosis of epithelial cells in bronchioles and pulmonary parenchyma, bronchointerstitial pneumonia with syncytia, hyperplasia of type II pneumocytes. Neutrophilic exudate	Acute severe mucohemorrhagic colitis; mucosal ulceration; compensatory hyperplasia, hemorrhages, microabscesses, vasculitis. Mild atrophic enteritis of small intestine		Mesenteric lymph nodes depletion and necrosis, moderate BALT hyperplasia
**Equine**	**Horse**		Betacoronavirus	Equine coronavirus (ECoV)	Systemic		Pulmonary congestion and edema	Necrotizing jejunitis and ileitis with pseudomembranes formation and crypt microabscesses, microthrombosis, crypt enterocytes intracytoplasmic inclusion bodies, hemorrages.haemorrhages. Ulceration of oral mucosa and tongue	Cerebral cortex Alzheimer Type II astrocytosis	Lymphocytolysis of Peyer's patches. Widespread petechial hemorrhages in the thymus
	**Donkey**		Betacoronavirus	Equine coronavirus (ECoV)	Systemic					Lymphocytolysis of Peyer's patches
	**Swine**	Transmissible	Alphacoronavirus	Transmissible	Localized			Ascites, severe atrophic		

	Gastroenteritis (TGE)		GastroEnteritis virus (TGEV)				jejunitis and ileitis, crypts hyperplasia		
**Host species**	**Name of disease/conditio n**	**Viral genus, subgenus**	**Virus strains/name**	**Main extension of disease**	**Main involved tissues and associated lesions**				
					**Upper respiratory tract**	**Lower respiratory tract**	**Alimentary system**	**Nervous system**	**Hematopoietic and lymphatic systems/ Urinary system**
**Swine**	Porcine Epidemic Diarrhea (PED)	Alphacoronavirus	Porcine Epidemic Diarrhea virus (PEDV)	Localized			Acute severe atrophic jejunitis, crypt hyperplasia, enterocytes syncytia and necrosis, attenuation and cytoplasmic vacuolation of superficial enterocytes. Crypt epithelial cells hyperplasia		
		Deltacoronavirus	Porcine Deltacorona virus (PDCoV)	Localized		Mild multifocal bronchial-to-bronchiol ar-centric areas of non-suppurative interstitial pneumonia	Acute severe atrophic jejunitis and ileitis, epithelial syncytia. Villous atrophy, attenuation, blunting and fusion, vacuolated enterocytes in cecum and colon, crypts hyperplasia		
		Alphacoronavirus	Swine Acute Diarrhea Syndrome coronavirus (SADS-CoV)	Localized			Acute severe atrophic enteritis (small intestine)		
	Porcine Hemagglutinati ng Encephalomyelit is (PHE)	Betacoronavirus	PHE virus	Systemic			Myenteric plexus degeneration (stomach-pyloric glandular area)	Replication and damage of sensory neurons (peripheral ganglia and CNS), perivascular cuffing around Meissner and Auerbach ganglia in the muscle layer of the stomach, ganglioneuritis in the	

									trigeminal ganglion, non-suppurative encephalomyelitis. Satellitosis and gliosis in brainstem (in piglets)	
			Alphacoronavirus	Porcine Respiratory Coronavirus (PRCV)	Localized		Interstitial pneumonia			
	**Host species**	**Name of disease/condition**	**Viral genus, subgenus**	**Virus strains/name**	**Main extension of disease**	**Main involved tissues and associated lesions**				
						**Upper respiratory tract**	**Lower respiratory tract**	**Alimentary system**	**Nervous system**	**Hematopoietic and lymphatic systems/ Urinary system**
**Wild felids**	**Cheetah**	Feline Infectious Peritonitis (FIP)		Feline coronavirus	Systemic			Gastric ulcers, erosive/necrotizing enteritis		Multifocal necrosis of spleen, thymus and lymph nodes
	**Mountain lion**	Feline Infectious Peritonitis (FIP)		Feline coronavirus	Systemic			Muscular necrosis and vasculitis in the intestine	Vacuolation of the neuropil with occasional axonal swelling of the posterior colliculus	
	**African lion**	Feline Infectious Peritonitis (FIP)		Feline coronavirus	Systemic				Dilated perivascular spaces mainly in the white matter	
	**Wildcat**			Feline coronavirus	Systemic		Patchy consolidation of the lungs	Thickening of the serosa surface of the intestine by fibrin and leucocytic inflammation	Vascular lesions in the meninges	
**Wild ruminants**	**Several species of deer**			BCoV-like coronavirus	Localized			Gastroenteritis		
	**Waterbuck antelope**	Winter dysentery		CoVs antigenically indistinguishable from BCoV	Localized			Gastroenteritis		
	**Giraffe**			BCoV-like coronavirus	Localized			Gastroenteritis		
	**Llama**		Betacoronavirus, Embecovirus	BCoV-like coronavirus	Localized			Gastroenteritis		

	**Alpaca**		Betacoronavirus, Embecovirus	Alpaca (beta) coronavirus (ACoV)	Systemic		Respiratory disease, undescribed lesions	Gastroenteritis		
			Alphacoronavirus, Duvinacovirus	Alpaca (alpha) coronavirus (ACoV)	Localized		Respiratory disease, undescribed lesions			
	**Dromedary camel**		Betacoronavirus, Embecovirus	Dromedary camel coronavirus UAE- HKU-23 (DcCoV UAE-HKU23)	Localized			Gastroenteritis		
			Betacoronavirus, Merbecovirus	MERS-related CoV	Localized	Mild upper respiratory tract infection				
	**Host species**	**Name of disease/conditio n**	**Viral genus, subgenus**	**Virus strains/name**	**Main extension of disease**	**Main involved tissues and associated lesions**				
						**Upper respiratory tract**	**Lower respiratory tract**	**Alimentary system**	**Nervous system**	**Hematopoietic and lymphatic systems/ Urinary system**
**Wild ruminants**	**Water buffalo**		Betacoronavirus, Embecovirus	Bubaline coronavirus (BuCoV)	Localized			Severe gastroenteritis, with congestion and hemorrhages		Enlargement of the mesenteric lymph nodes
	**Elk (wapiti)**			BCoV-like coronavirus	Systemic		Interstitial pneumonia with syncytia formation	Enteritis with syncytia formation		
	**Musk oxen, Sitatunga, Wisent, Hymalayan tahr, Nyala**	Winter dysentery		BCoV-like coronavirus	Localized			Severe bloody diarrhea		
**Other wild animals**	**American and European mink**	Mink epizootic catarrhal gastroenteritis (ECG)	Alphacoronavirus, Minacovirus	Mink coronavirus 1	Localized			Severe acute catarrhal gastroenteritis		
				SARS-CoV-2	Localized		Diffuse interstitial pneumonia with hyperemia, alveolar damage, and atelectasis			

	**Host species**	**Name of disease/condition**	**Viral genus, subgenus**	**Virus strains/name**	**Main extension of disease**	**Main involved tissues and associated lesions**				
						**Upper respiratory tract**	**Lower respiratory tract**	**Alimentary system**	**Nervous system**	**Hematopoietic and lymphatic systems/ Urinary system**
	**European and Italian wolf**			Canine enteric coronaviruses	Localized			No lesions described		
	**Red fox**			SARS-like CoV	Systemic			No lesions described		
				Canine CoV	Localized			Mild, self-limiting enteritis		
	**Raccoon**		Alphacoronavirus-1	SARS-like CoV	Systemic		Mild bronchopneumonia	Fibrinous gastroenteritis of small intestine, and diffuse blunting and fusion of intestinal villi		
	**Eurasian otter**			Canine CoV	Localized			Mild, self-limiting enteritis		
	**Common genet**			Canine CoV	Localized			Mild, self-limiting enteritis		
	**Masked palm civet**			SARS-like CoV	NR			No lesions described		
	**Spotted hyena**		Alphacoronavirus	Feline CoV type II and Canine CoV	NR			No lesions described		
	**Silver-backed jackal**		Alphacoronavirus		NR			No lesions described		
**Other wild animals**	**European hedgehog**			Hedgehog coronavirus 1 (Erinaceus CoV, EriCoV)	NR			No lesions described		
	**Amur hedgehog**			Erinaceus amurensis hedgehog coronavirus HKU31 (Ea-HedCoV HKU31)	NR			No lesions described		
	**Asian house shrew**		Alphacoronavirus	Wénchéng shrew coronavirus (WESV)	NR			No lesions described		

**NHP**	**Chimpanze (wild)**		Betacoronavirus 1	Human coronavirus (HCoV) OC43	Localized		Respiratory disease, undescribed lesions			
**Marine mammals**	**Beluga whale**			Beluga whale coronavirus SW1 (BWCoV-SW1)	Localized		Respiratory disease, undescribed lesions			
	**Indo-Pacific bottlenose dolphin**			Bottlenose dolphin CoV (BdCoV) HKU22	NR			No lesions described		
**Lab animals**	**Mouse**		Betacoronavirus, Embecovirus	Mouse hepatitis virus (MHV) polytropic	Systemic	Necrosis of olfactory epithelium	Interstitial pneumonia		Nasoencephalitis, necrotizing meningoencephalitis, demyelination, axonal loss, syncytia	Necrosis of spleen, lymph nodes, thymus, GALT, bone marrow; syncytia of parenchymal and endothelial cells
				Mouse hepatitis virus (MHV) enterotropic	Systemic			Necrotizing enterocolitis, villous attenuation, syncytia of enterocytes and endothelial cells, eosinophilic intracytoplasmic inclusion	Encephalitis	Lymphocytic syncytia in mesenteric lymph nodes
	**Rat**		Betacoronavirus, Embecovirus	Sialodacryo-adenitis virus (SDAV)	Systemic	Necrotizing rhinitis, laryngitis	Necrotizing tracheitis, bronchitis and bronchiolitis, interstitial pneumonia	Necrotizing sialoadenitis	Necrotizing encephalitis	
	**Host species**	**Name of disease/condition**	**Viral genus, subgenus**	**Virus strains/name**	**Main extension of disease**	**Main involved tissues and associated lesions**				
						**Upper respiratory tract**	**Lower respiratory tract**	**Alimentary system**	**Nervous system**	**Hematopoietic and lymphatic systems/ Urinary system**
**Lab animals**	**Rat**			Parker's rat coronavirus (PRC)	Systemic		Necrotizing tracheitis, interstitial pneumonia			
	**Guinea pigs**	Coronavirus-like syndrome	Unknown		Localized			Necrotizing enteritis, blunting and fusion of villi, syncytia of enterocytes		
**Fish**	**White bream**		Bafinivirus	White bream virus	Systemic		Gills hemorrhages			Congestion and hemorrhages

	**Cyprinids**		Bafinivirus		Systemic		Gills hemorrhages			
**Reptiles**	**Nile crocodiles**		Unknown		NR			No lesions described		
**Avian species**	**Chicken**	Infectious bronchitis	Gammacoronavirus, Igacovirus	Infectious bronchitis virus (mainly M41) (IBV)	Localized	Catarrhal rhinitis, laryngitis	Catarrhal tracheitis and bronchitis, catarrhal pneumonia, catarrhal or fibrinous airsacculitis		Perivascular lymphocytic cuffing, degenerated neurons, satellitosis and neuronophagia	Interstitial lymphocytic and heterophilic nephritis, hemorrhages, tubular degeneration and necrosis, edema of Bowman's capsule, and dilated renal tubules with urate crystals and casts
**Avian species**	**Pheasant**	Pheasant corona-virus-associated nephritis or Pheasant coronavirus nephritis or Pheasant urolithiasis	Gammacoronavirus, Igacovirus	Pheasant CoV (IBV-like)	Localized		Tracheitis			Interstitial nephritis
**Avian species**	**Pigeon**		Gammacoronavirus, Igacovirus	Pigeon CoV	Systemic		Lung lesions	Pancreatitis		
**Avian species**	**Turkey**		Gammacoronavirus	Turkey CoV	Localized			Severe enteritis		
**Avian species**	**Guinea fowl**		Gmmacoronavirus	Guinea fowl CoV	Localized			Severe enteritis		
**Avian species**	**Quail**		Gammacoronavirus	Quail CoV (IBV-like)	Localized	Laryngitis	Bronchitis and pneumonia			Renal disease, undescribed lesions
**Avian species**	**Green-cheeked amazon parrot**		Gammacoronavirus		Localized			Dilation of proventricolus		
	**Host species**	**Name of disease/condition**	**Viral genus, subgenus**	**Virus strains/name**	**Main extension of disease**	**Main involved tissues and associated lesions**				
						**Upper respiratory tract**	**Lower respiratory tract**	**Alimentary system**	**Nervous system**	**Hematopoietic and lymphatic systems/ Urinary system**
**Avian species**	**Greylag geese**		Gammacoronavirus		NR			No lesions described		
**Avian species**	**Mallard duck**		Gammacoronavirus		NR			No lesions described		
